# Exposure to Fine Particulate Air Pollution Causes Vascular Insulin Resistance by Inducing Pulmonary Oxidative Stress

**DOI:** 10.1289/EHP212

**Published:** 2016-04-29

**Authors:** Petra Haberzettl, Timothy E. O’Toole, Aruni Bhatnagar, Daniel J. Conklin

**Affiliations:** Diabetes and Obesity Center, Institute of Molecular Cardiology, School of Medicine, University of Louisville, Louisville, Kentucky, USA

## Abstract

**Background:**

Epidemiological evidence suggests that exposure to ambient air fine particulate matter (PM2.5) increases the risk of developing type 2 diabetes and cardiovascular disease. However, the mechanisms underlying these effects of PM2.5 remain unclear.

**Objectives:**

We tested the hypothesis that PM2.5 exposure decreases vascular insulin sensitivity by inducing pulmonary oxidative stress.

**Methods:**

Mice fed control (10–13% kcal fat) and high-fat (60% kcal fat, HFD) diets, treated with 4-hydroxy-2,2,6,6-tetramethylpiperidine-1-oxyl (TEMPOL) or mice overexpressing lung-specific extracellular superoxide dismutase (ecSOD) were exposed to HEPA-filtered air or to concentrated PM2.5 (CAP) for 9 or 30 days, and changes in systemic and organ-specific insulin sensitivity and inflammation were measured.

**Results:**

In control diet–fed mice, exposure to CAP for 30 days decreased insulin-stimulated Akt phosphorylation in lung, heart, and aorta but not in skeletal muscle, adipose tissue, and liver and did not affect adiposity or systemic glucose tolerance. In HFD-fed mice, 30-day CAP exposure suppressed insulin-stimulated endothelial nitric oxide synthase (eNOS) phosphorylation in skeletal muscle and increased adipose tissue inflammation and systemic glucose intolerance. In control diet–fed mice, a 9-day CAP exposure was sufficient to suppress insulin-stimulated Akt and eNOS phosphorylation and to decrease IκBα (inhibitor of the transcription factor NF-κB levels in the aorta. Treatment with the antioxidant TEMPOL or lung-specific overexpression of ecSOD prevented CAP-induced vascular insulin resistance and inflammation.

**Conclusions:**

Short-term exposure to PM2.5 induces vascular insulin resistance and inflammation triggered by a mechanism involving pulmonary oxidative stress. Suppression of vascular insulin signaling by PM2.5 may accelerate the progression to systemic insulin resistance, particularly in the context of diet-induced obesity.

**Citation:**

Haberzettl P, O’Toole TE, Bhatnagar A, Conklin DJ. 2016. Exposure to fine particulate air pollution causes vascular insulin resistance by inducing pulmonary oxidative stress. Environ Health Perspect 124:1830–1839; http://dx.doi.org/10.1289/EHP212

## Introduction

Recent studies have suggested that urbanization and the associated increase in air pollution could be a contributing factor to the worldwide increase in the incidence of diabetes (Bhatnagar 2009; Rao et al. 2015). Because the rates of obesity and type 2 diabetes (T2D) have increased significantly in only a few generations [Centers for Disease Control and Prevention (CDC) 2016], it is likely that this increase is attributable, at least in part, to environmental factors such as lifestyle choices, community environment, and exposure to polluted air, rather than to population-wide genetic changes. In line with this view, several epidemiological studies have shown that exposure to air pollution increases the risk of T2D (Chen et al. 2013; Coogan et al. 2012; Eze et al. 2015; Krämer et al. 2010; Park et al. 2015; Pearson et al. 2010). Exposure to ambient air pollution has also been linked to an increase in diabetes-associated mortality (Brook et al. 2013a; Pope et al. 2015), exacerbation of cardiometabolic disorders (Pope et al. 2015), and poor metabolic control in individuals with (Tamayo et al. 2014) or without (Brook et al. 2013b) diabetes. An analysis of individual U.S. counties found a significant positive association between ambient levels of airborne fine particulate matter (≤ 2.5 μm, PM_2.5_) and the prevalence of T2D, but not obesity (Pearson et al. 2010), suggesting that exposure to air pollution may be an obesity-independent T2D risk factor. Taken together, these studies raise the possibility that the current epidemic of T2D may be a result of recurrent exposure to high levels of ambient air pollutants.

Experimental data from animal studies support the idea that there is a biologically plausible link between PM_2.5_ exposure and the development of diabetes. Specifically, it has been reported that in mice fed a high-fat diet (HFD), prolonged exposure to concentrated ambient PM_2.5_ increases systemic insulin resistance and visceral adiposity (Sun et al. 2009). Long-term exposure to concentrated PM_2.5_ was found to induce systemic insulin resistance in mice, even in the absence of an HFD (Xu et al. 2011). Although these studies found that PM_2.5_ induces metabolic dysfunction, it remains unclear how this defect develops and whether the systemic effects of PM_2.5_ are linked to and preceded by changes specific to cardiovascular tissues. Understanding the processes by which PM_2.5_ exposure affects the cardiovascular system is important because PM_2.5_ exposure has been associated with an increase in cardiovascular disease (CVD) risk (Brook et al. 2010), which may be related to defects in insulin signaling in cardiovascular tissues, independent of systemic insulin resistance.

In models of diet-induced obesity, vascular insulin resistance precedes the development of insulin resistance in skeletal muscle, liver, and adipose tissue (Kim et al. 2008), suggesting that the development of “early” vascular insulin resistance plays a critical, if not an essential, role in the subsequent development of systemic glucose intolerance. Vascular dysfunction is an early event in the development of diabetes (Kim et al. 2006), and defective insulin signaling in the vasculature has been reported to be sufficient and necessary for the development of systemic insulin resistance (Kubota et al. 2011). Nevertheless, it remains unclear how inhaled PM_2.5_ affects insulin signaling in blood vessels. Therefore, we examined the effects of PM_2.5_ exposure to determine whether changes in vascular insulin signaling are secondary to diet-induced changes and an increase in systemic or pulmonary oxidative stress. Oxidative stress plays a well-described role in mediating the toxicity of PM_2.5_, and mice deficient in the NADPH oxidase subunit p47(phox), which generates reactive oxygen species (ROS), are protected against the effects of PM_2.5_ exposure on systemic insulin resistance (Xu et al. 2010).

We found that short-term inhalation of concentrated ambient PM_2.5_ (CAP) induced vascular insulin resistance independent of dyslipidemia, obesity, and systemic inflammation and that PM_2.5_-induced vascular insulin resistance could be mitigated by increasing the removal of superoxide in the lung. These findings reveal a novel link between pulmonary oxidative stress and vascular insulin resistance—a link that may explain how air pollution increases the risk of developing both cardiovascular and metabolic disease.

## Methods

### Animal Studies

**Figure 1:**
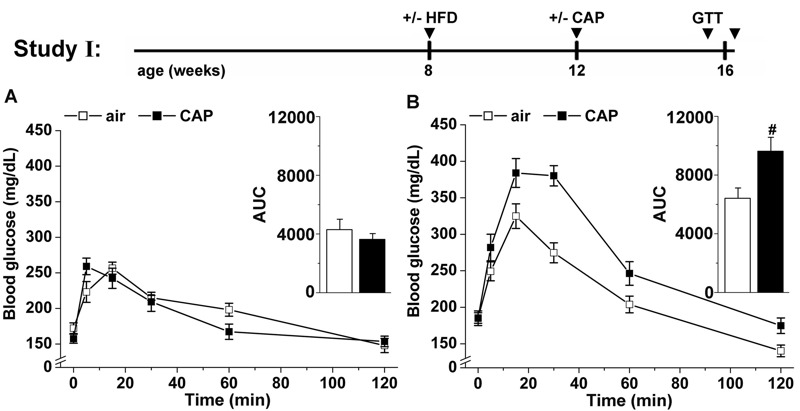
Effects of concentrated fine particulate matter (CAP) exposure on systemic glucose homeostasis. Mice maintained on control diet (13% kcal fat) or placed on a high-fat diet (HFD, 60% kcal fat) were exposed to air or CAP for 30 days (Study I). After 25 days of exposure, systemic glucose tolerance was tested in both control (*A*) and HFD-fed (*B*) mice. The total excursion of glucose in the blood was calculated by integrating the area under the curve (AUC, inset). Data are the mean ± SE. ^#^ *p* < 0.05 air versus CAP; *n* = 8. GTT, glucose tolerance test.

**Figure 2:**
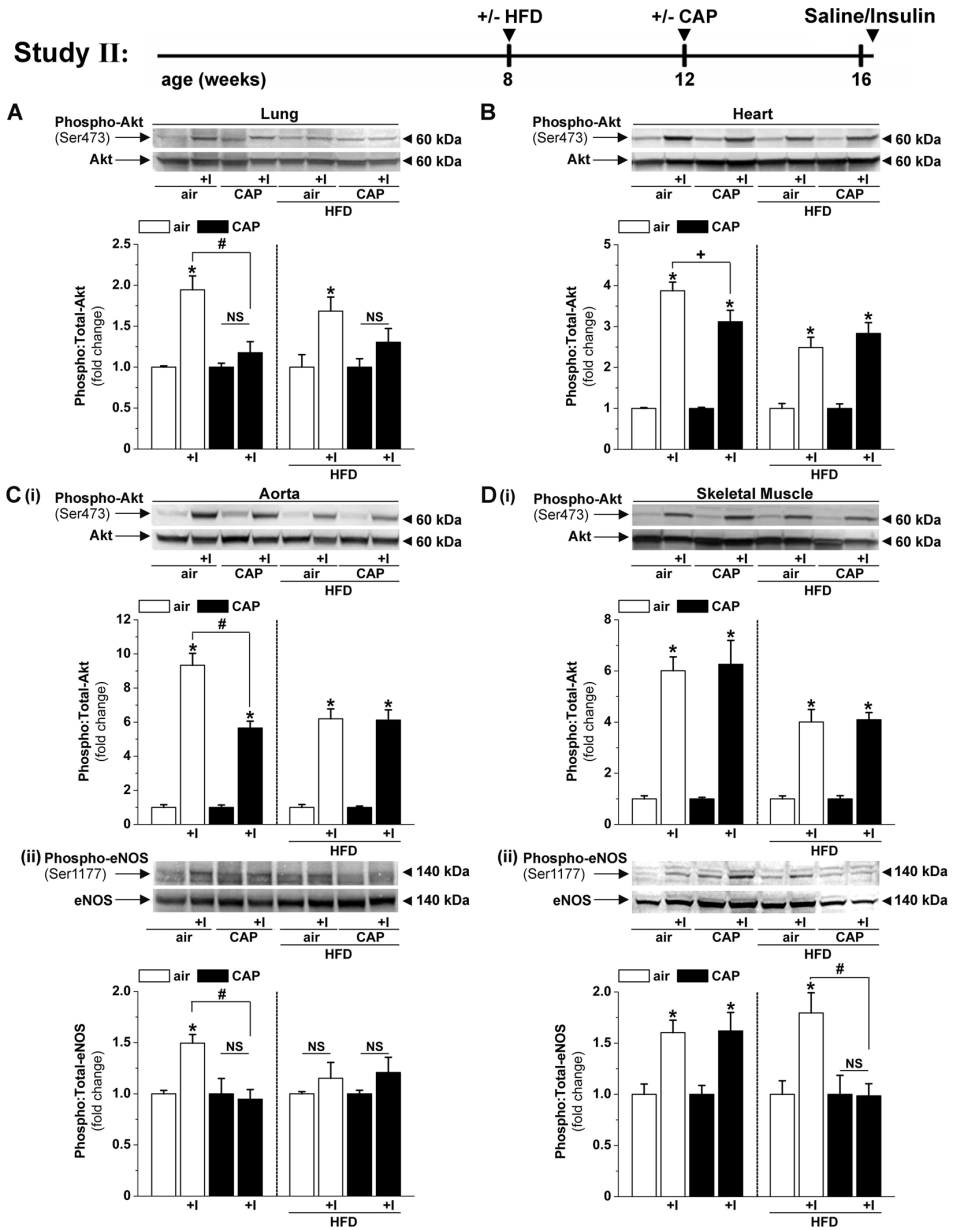
Effects of concentrated fine particulate matter (CAP) exposure on organ-specific insulin sensitivity. Western blot analysis of Akt phosphorylation in lung (*A*) and heart (*B*) and phosphorylation of Akt (*i*) and eNOS (*ii*) in aorta (*C*) and skeletal muscle (*D*) in mice injected with saline or insulin (1.5 U/kg). Mice fed control diet (10% kcal fat) or placed on a high-fat diet (HFD, 60% kcal fat) were exposed to air or CAP for 30 days (Study II). Data are the mean ± SE normalized to controls. NS, not significant. *p < 0.05 saline vs. insulin; ^#^ *p* < 0.05, ^+^ *p* < 0.1 air versus CAP; *n* = 4.

**Figure 3:**
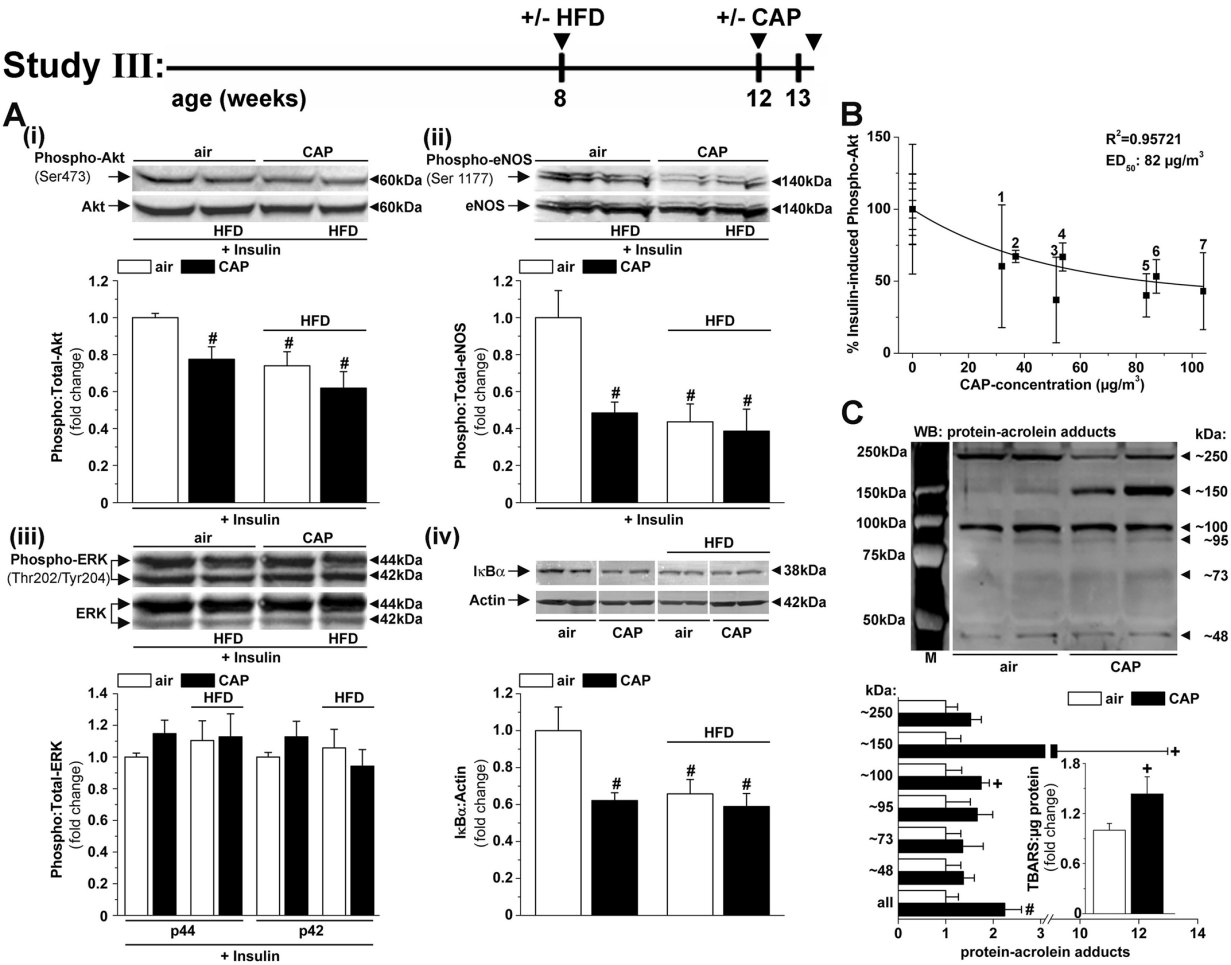
Concentrated fine particulate matter (CAP) exposure induces aortic insulin resistance and vascular inflammation. (*A*) Western blot analysis of the insulin-stimulated (100 nM, 15 min) phosphorylation of Akt (*i*), eNOS (*ii*) and ERK (*iii*) and the abundance of IκBα (*iv*) in aortas isolated from mice maintained on control diet (13% kcal fat) or placed on a high-fat diet (HFD, 60% kcal fat) that were exposed to air or CAP for 9 days (Study III). A continuous Western blot to detect the abundance of IκBα is shown in Figure S4A. Data are the mean ± SE normalized to controls (^#^ *p* < 0.05 vs. air-exposed control diet-fed mice; phospho-Akt, *n* = 8; phospho-eNOS, *n* = 5; phospho-ERK, *n* = 6; IκBα, *n* = 4). (*B*) Dose dependency of CAP-induced vascular insulin resistance was analyzed in insulin-stimulated aortas isolated from mice exposed to air or CAP for 9 or 30 days. For each exposure performed between 2010 and 2013 (see Table S4), the extent of insulin-induced Akt phosphorylation in the aorta was measured by Western analysis as described. Data are shown as discrete points of insulin-induced Akt phosphorylation (mean ± SE, in percent of air-exposed controls) and the cumulative CAP dose for each exposure, and the curve is a best fit of a first-order exponential equation [(*y* = 39.0**+ 60.9**exp (–*x*/49.2); *y* = percent insulin-induced phospho-Akt, *x* = CAP concentration in micrograms per cubic meter] to the data. (*C*) Western blot analysis of the plasmatic abundance of protein–acrolein adducts (loading controls are shown in Figure S4C) and plasma TBARS levels (*C*, inset) in mice exposed to air or CAP for 9 days. Data are mean ± SE (^#^ *p* < 0.05, ^+^ *p* < 0.1 air vs. CAP; *n* = 5). ED_50_, median effective dose; TBARS, thiobarbituric acid reactive substances.

**Figure 4:**
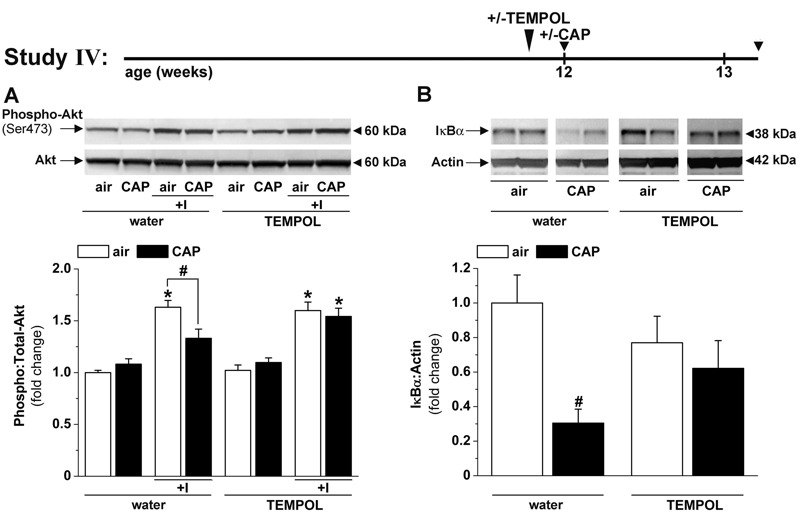
TEMPOL treatment prevents concentrated fine particulate matter (CAP)-induced vascular insulin resistance and inflammation. Western blot analysis of (*A*) the insulin-stimulated phosphorylation of Akt and (*B*) the abundance of IκBα in aortas of mice treated with water or TEMPOL (1 mM, in drinking water) exposed to air or CAP for 9 days (Study IV). The continuous IκBα Western blot is shown in Figure S4B. Data are the mean ± SE normalized to controls. *p < 0.05 control versus insulin; ^#^ *p* < 0.05 air versus CAP. Phospho-Akt, *n* = 5–10; IκBα, *n* = 4–5.

**Figure 5:**
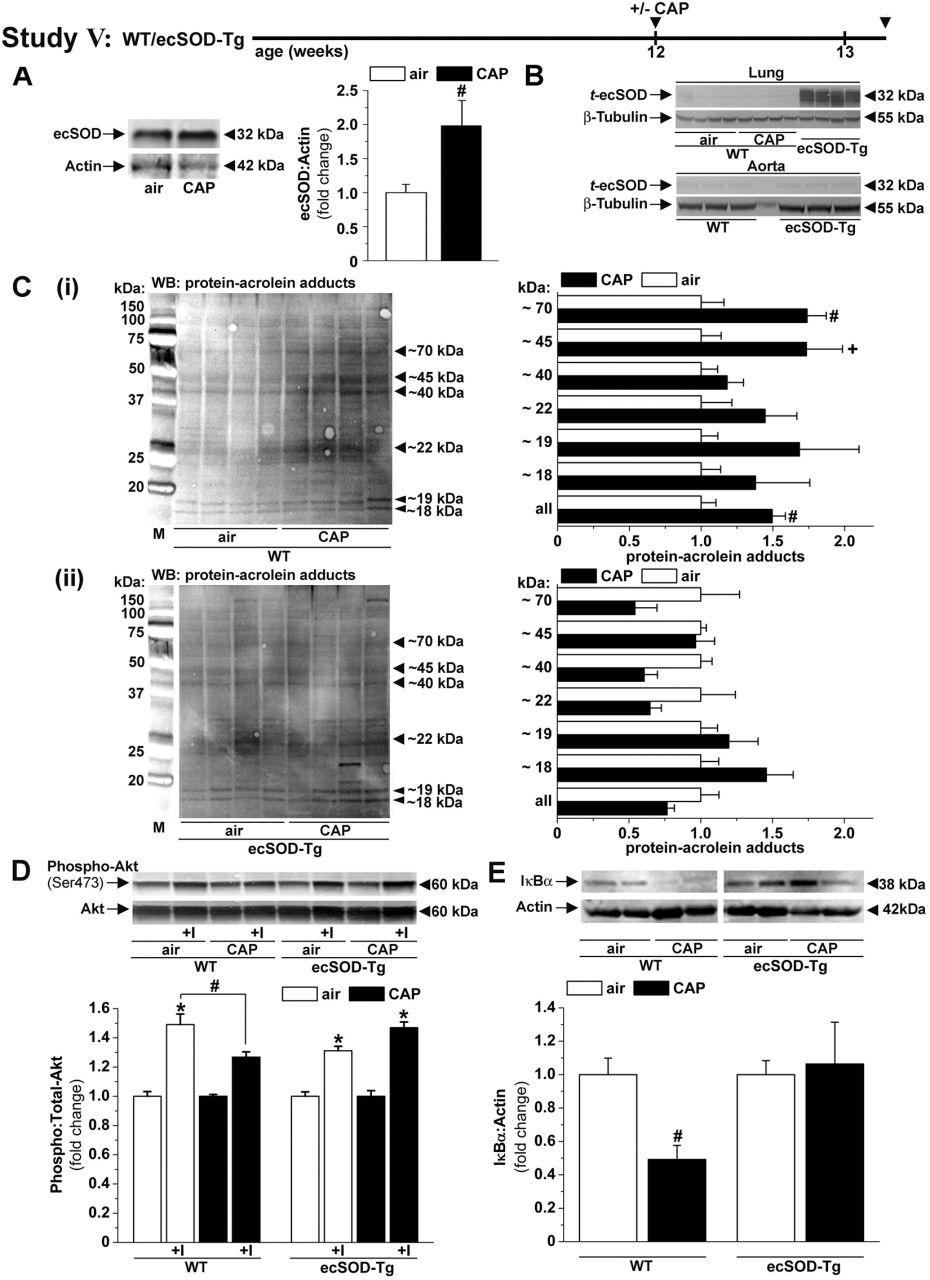
Concentrated fine particulate matter (CAP)-induced vascular insulin resistance and inflammation are prevented in lung-specific ecSOD transgenic (ecSOD-Tg) mice. (*A*) Western blot analysis of the pulmonary ecSOD protein abundance (*n* = 4) in wild-type (WT) mice exposed to air or CAP for 9 days. (*B*) Western blots of the transgene ecSOD (*t-*ecSOD) protein abundance in lung and aorta isolated from WT mice exposed to air or CAP for 9 days and from ecSOD-Tg mice (Study V). Western blot analysis of the (*C*) pulmonary abundance of protein–acrolein adducts (loading controls are shown in Figure S4D, *n* = 4–5), (*D*) aortic insulin-stimulated Akt phosphorylation (*n* = 5–8) and (*E*) aortic IκBα abundance (*n* = 8–12) in WT and ecSOD-Tg mice exposed to air or CAP for 9 days. Data are the mean ± SE normalized to controls. *p < 0.05 control versus insulin; ^#^ *p* < 0.05 and ^+^ *p* < 0.1 air versus CAP.

Male C57BL/6 mice, approximately 12 weeks old, fed a control diet (10–13% kcal fat) or a high-fat diet (HFD; 60% kcal fat, Figures 1–3, Studies I–III) were exposed to high-efficiency particulate arrestance (HEPA)-filtered air or to concentrated ambient PM_2.5_ (CAP, 6 hr/day) for 9 or 30 consecutive days as described in the Supplemental Material, “Methods.” In addition, mice treated with 4-hydroxy-2,2,6,6-tetramethylpiperidine-1-oxyl (TEMPOL, Figure 4, Study IV) and mice transgenic (Tg) for lung-specific extracellular superoxide dismutase (ecSOD, Figure 5, Study V) fed control diet (13% kcal fat) were exposed to air or to CAP (6 hr/day) for 9 consecutive days. Body weight was measured weekly, and a glucose tolerance test (GTT) was performed (day 25, Study I). For the GTT, mice fasted for 6 hr were injected with glucose [1 g/kg body weight, intraperitoneally (i.p.)], and their blood glucose levels were monitored (ACCU-CHECK, Aviva; Roche). Finally, after 6 hr food withdrawal, blood and organs were collected. Plasma was used for biochemical analysis as described previously (Conklin et al. 2009) or using commercial kits (Mouse Multi/Singleplex-adipokine/adiponectin, Luminex 200, Millipore; TBARS, ZeptoMetrix). Insulin signaling was examined in organs of mice injected (i.p., 0.1 mL, 15 min, Study II) with saline or insulin (Humulin-RP, Eli-Lilly, 1.5 U/kg), or in isolated aorta (Studies III–V) (Haberzettl et al. 2012) stimulated *ex vivo* with vehicle or insulin (100 nM, 15 min). Mice were treated humanely according to the APS’s Guiding Principles in the Care and Use of Animals following protocols approved by the University of Louisville Institutional Animal Care and Use Committee (IACUC).

### Immunoblotting

Western blot analyses were performed using indicated antibodies as described in the Supplemental Material, “Methods.”

### Statistical Analyses

Data are presented as the mean ± SE. Statistical significance (*p* < 0.05) was determined using an unpaired Student’s *t-*test or one-way analysis of variance (ANOVA) with a Bonferroni post-hoc test (SigmaStat, SPSS) where appropriate. To analyze the dose–response of CAP concentration and aortic insulin signaling, a nonlinear first-order exponential equation [(*y* = *y*_0_ + *a*_1_ exp (–*x*/*t*_1_)], chosen based on the regression coefficient *R* and on a chi-squared test, was fitted to the data.

## Results

### Effects of CAP Exposure on Systemic Glucose Homeostasis

To examine how PM_2.5_ exposure affects systemic insulin resistance, we exposed control and HFD-fed mice to air or CAP for 30 days (Study I, Figure 1) and measured systemic glucose tolerance by GTT and calculated homeostatic model assessment scores for insulin resistance (HOMA-IR) and β cell function (HOMA-β), which reflect systemic insulin resistance and insulin release, respectively. We found that in control diet–fed mice, CAP exposure led to neither systemic glucose intolerance (Figure 1A) nor changes in body weight, fasting blood glucose, plasma insulin levels, or the HOMA-IR and HOMA-β scores (Table 1) indicating that the mice were not insulin resistant or glucose intolerant. However, CAP-exposed mice had higher plasma levels of total and low-density lipoprotein (LDL) cholesterol and a lower high-density lipoprotein (HDL):LDL ratio than air-exposed mice (Table 1). CAP exposure did not affect the plasma levels of major adipokines (adiponectin, leptin, resistin), cytokines (TNF-α, IL-6), or markers of liver [total protein, albumin, alanine aminotransferase (ALT), aspartate aminotransferase (AST)] and muscle [creatinine kinase, (CK)] (Table 1). Moreover, there was no increase in pulmonary inflammation as indicated by unchanged levels of IL-1β, IL-6, MCP-1 and TNF-α mRNAs in the lungs (see Table S3). Collectively, these data indicate that exposure to CAP for 30 days induced mild dyslipidemia but not systemic insulin resistance, obesity, lung inflammation, or overt muscle or hepatic injury.

**Table 1:** Systemic effects of CAP exposure for 30 days (Study I).

Parameter, unit, *n*	Air	CAP	*p*	Air + HFD	CAP + HFD	*p*
Body weight, g, 8	27.88 ± 0.58	27.76 ± 0.53	0.882	31.69 ± 0.87	32.25 ± 0.92	0.665
Glucose, mg/dL, 8	151 ± 9	162 ± 9	0.374	175 ± 11	171 ± 10	0.781
Insulin, ng/mL, 4–5	0.38 ± 0.03	0.36 ± 0.03	0.660	0.55 ± 0.01	0.69 ± 0.04	0.019
HOMA-IR, 4–5	4.2 ± 0.3	3.9 ± 0.4	0.609	6.4 ± 0.3	8.6 ± 0.6	0.014
HOMA-β (%), 4–5	42.5 ± 3.7	44.9 ± 7.4	0.780	56.5 ± 4.6	66.2 ± 12.4	0.490
Adiponectin, ng/mL, 5	13.38 ± 3.01	11.32 ± 1.55	0.559	11.34 ± 1.56	10.06 ± 2.87	0.704
Leptin, ng/mL, 5	0.39 ± 0.03	0.35 ± 0.04	0.385	0.77 ± 0.21	0.78 ± 0.22	0.975
Resistin, ng/mL, 5	3.01 ± 0.56	3.12 ± 0.58	0.899	3.24 ± 0.28	3.28 ± 0.40	0.928
TNF-α, pg/mL, 5	4.24 ± 0.80	2.60 ± 0.35	0.108	4.09 ± 0.78	4.10 ± 1.00	0.991
IL-6, pg/mL, 5	17.29 ± 4.03	16.89 ± 2.10	0.933	13.98 ± 0.81	9.98 ± 2.88	0.230
TG, mg/dL, 6–8	19.44 ± 0.73	19.11 ± 3.36	0.911	17.50 ± 2.29	21.52 ± 3.19	0.323
Cholesterol, mg/dL, 6–8	79.56 ± 2.42	113.09 ± 5.53	0.001	129.96 ± 3.76	154.69 ± 8.74	0.021
HDL, mg/dL, 6–8	57.40 ± 2.07	65.70 ± 5.93	0.187	95.14 ± 5.27	108.02 ± 5.60	0.116
LDL, mg/dL, 6–8	10.38 ± 0.58	14.14 ± 1.53	0.024	19.79 ± 2.36	17.36 ± 1.03	0.360
HDL:LDL, 6–8	5.36 ± 0.11	4.73 ± 0.24	0.030	5.05 ± 0.35	6.34 ± 0.44	0.038
TP, g/dL, 5–8	5.22 ± 0.13	5.32 ± 0.17	0.653	5.47 ± 0.19	5.64 ± 0.21	0.544
Albumin, g/dL, 5–8	3.62 ± 0.15	3.56 ± 0.14	0.765	3.36 ± 0.07	3.70 ± 0.16	0.068
Albumin: TP, 5–8	0.69 ± 0.02	0.67 ± 0.01	0.276	0.62 ± 0.02	0.66 ± 0.02	0.115
ALT, U/L, 5–8	30.60 ± 5.70	30.91 ± 2.98	0.962	30.03 ± 3.35	27.84 ± 3.21	0.644
AST, U/L, 4–8	48.39 ± 6.71	62.33 ± 13.10	0.380	72.29 ± 11.52	72.48 ± 4.96	0.988
CK, U/L, 4–8	141.22 ± 73.49	171.53 ± 83.83	0.797	245.98 ± 43.10	241.99 ± 25.24	0.937
Notes: ALT, alanine aminotransferase; AST, aspartate aminotransferase; CAP, concentrated fine particulate matter; CK, creatinine kinase; HDL, high-density lipoprotein cholesterol; HOMA-β (= 20 × fasting plasma insulin levels [mU/L]/fasting blood glucose [mmol/L] – 3.5), %; HOMA-IR (= fasting blood glucose [mmol/L] × fasting plasma insulin levels [mU/L]/22.5); IL-6, interleukin 6; LDL, low-density lipoprotein cholesterol; TG, triglycerides; TNF-α, tumor necrosis factor α; TP, total protein. Blood and plasma parameters were measured in control or high-fat diet (HFD)-fed mice exposed for 30 days to air or CAP. Data represent the mean ± SE; *p*, air versus CAP.						

The absence of CAP-induced changes in glucose tolerance and HOMA-IR scores suggests that PM exposure did not induce systemic insulin resistance in the mice that were fed the control diet; however, to test whether PM_2.5_ exposure exacerbates pre-existing insulin resistance, we examined the effects of CAP exposure in mice with insulin resistance induced by HFD (Study I; Figure 1). We found that CAP exposure significantly exacerbated both glucose intolerance [Figure 1B, see the increased area under the curve (AUC) for GTT, inset] and the HOMA-IR score in these mice (Table 1); however, CAP exposure did not affect the HOMA-β score, suggesting that pancreatic β-cell function was unaltered (Table 1). Exposure to CAP also increased the plasma levels of total cholesterol, although no change in the levels of adipokines, cytokines, or muscle or liver enzymes was observed in comparison with air-exposed, HFD-fed mice (Table 1). CAP exposure did not alter body weight gain or adiposity, but it did attenuate HFD-induced adipocyte hypertrophy, and it enhanced adipose tissue inflammation as indicated by increased levels of F4/80^+^-cells, crown-like structures (CLS), and TNF-α and MIP-1α mRNA in the adipose tissue (see Figure S1A–E). Nevertheless, no changes in proinflammatory mRNA levels in the lungs were apparent (see Table S3). These observations suggest that CAP exposure exacerbates HFD-induced systemic insulin resistance and adipose tissue inflammation independent of changes in adiposity, pulmonary inflammation, and systemic toxicity.

### Effects of CAP Exposure on Organ-Specific Insulin Sensitivity

Previous work has shown that in rodent models of diet-induced obesity, vascular insulin resistance (measured as decreased insulin-stimulated Akt and eNOS phosphorylation) is necessary and sufficient for, and thus precedes, the subsequent development of organ-specific and systemic insulin resistance (Kim et al. 2008; Kubota et al. 2011). Nevertheless, it is unknown whether CAP exposure induces vascular insulin resistance and whether CAP-induced vascular insulin resistance precedes organ-specific insulin resistance. Therefore, to examine the effects of CAP exposure on changes in organ-specific insulin sensitivity, we exposed control and HFD-fed mice to air or CAP for 30 days (Study II, Figure 2). In control diet–fed mice, CAP exposure reduced insulin-stimulated Akt phosphorylation in the lungs (Figure 2A), heart (Figure 2B), and aorta (Figure 2Ci), as well as eNOS phosphorylation in the aorta (Figure 2Cii), without affecting insulin signaling in the skeletal muscle (Figure 2D) or decreasing the abundance of the aortic or cardiac insulin receptor (data not shown). These results indicate that CAP exposure caused early decreases in insulin sensitivity in the lung, heart, and aorta, which precede the development of systemic insulin resistance (Figure 1A).

As expected, mice that were fed HFD showed decreased insulin-stimulated Akt phosphorylation in the heart, aorta, and skeletal muscle, but this defect was not further exacerbated by CAP exposure (Figure 2). In HFD-fed mice, CAP exposure did not further suppress insulin-stimulated eNOS phosphorylation in the aorta (Figure 2Cii), but it completely prevented insulin-stimulated eNOS phosphorylation in the skeletal muscle (Figure 2Dii). This decrease in total skeletal muscle eNOS phosphorylation (Figure 2Cii) was associated with an increase in whole-body insulin resistance (Figure 1B), suggesting that the CAP-induced increase in systemic glucose intolerance in HFD-fed mice might be related to an increase in endothelial insulin resistance in the skeletal muscle. CAP exposure decreased eNOS phosphorylation (likely an endothelial-specific event) without affecting Akt phosphorylation (Akt is ubiquitous), suggesting that CAP exposure specifically affects the endothelium. Moreover, because CAP exposure did not affect insulin-stimulated Akt phosphorylation in the adipose tissue or in the liver (data not shown), it appears that disruption of insulin signaling in the endothelium of the skeletal muscle is sufficient to account for the increase in systemic glucose intolerance in CAP-exposed mice.

### CAP Exposure Induces Early Vascular Insulin Resistance and Inflammation

Because the results of Studies I and II showed that exposure to CAP for 30 days impairs insulin signaling in multiple tissues, we asked whether a briefer exposure would induce cardiovascular insulin resistance. To address this question, we exposed control and HFD-fed mice to air or CAP for 9 days (Figure 3; Study III) and then measured insulin signaling in the aorta and heart *ex vivo* (to preclude systemic effects of insulin such as changes in blood flow and blood pressure). We found that a 9-day CAP exposure suppressed insulin-stimulated Akt phosphorylation in the heart (see Figure S2A). In the aorta, the 9-day CAP exposure decreased insulin-stimulated phosphorylation of Akt (Figure 3Ai) and eNOS (Figure 3Aii) but not ERK (Figure 3Aiii; see also Figure S2B). These changes were accompanied by a decrease in aortic IκBα levels (Figure 3Aiv). Collectively, these results indicate that CAP exposure activates the pro-inflammatory NFκBα pathway and that it selectively impairs the insulin-induced activation of the PI3K/Akt/eNOS pathway, but not the MAPK/ERK pathway.

Having found that a decrease in insulin-stimulated Akt phosphorylation in the aorta is a sensitive measure of CAP-induced injury, we examined the dose-dependence of this response. As shown in Figure 3B, we found a nonlinear relationship between CAP concentration and vascular insulin resistance with a median effective dose (ED_50_) of 82 μg/m^3^. This dose corresponds to a PM_2.5_ exposure level of approximately 20 μg/m^3^ for 24 hr, which is comparable to the CAP concentrations used in previous animal studies (Sun et al. 2009; Xu et al. 2011) and similar to the 24-hr average levels of PM_2.5_ in most major U.S. cities, which vary from 20 to 35 μg/m^3^ (Brook et al. 2010).

Because our results showed that CAP exposure attenuates aortic insulin signaling, we next examined whether this was accompanied by vascular dysfunction. We found that CAP exposure (for 9 or 30 days) did not induce frank endothelium dysfunction measured as acetylcholine-mediated relaxation in the aorta (Studies I and III; data not shown); however, CAP exposure for 9 days potentiated the contractile responses of the aorta to both phenylephrine (alpha-adrenergic agonist) and high potassium (non-receptor agonist) (see Figure S2Ci and Table S5), which may also reflect a diminished endothelium-dependent relaxation capacity (Walker et al. 1997). When the exposure duration was extended to 30 days, the early hypercontractile response switched to hypocontractility (see Figure S2Cii and Table S5), a condition already present in the aortas of HFD-fed mice (see Figure S2Ci,ii and Table S5). These data indicate that even in the absence of HFD, short-term CAP exposure results in early vascular dysfunction.

### Role of Systemic Oxidative Stress in Mediating CAP-Induced Vascular Insulin Resistance and Inflammation

To examine the mechanism of CAP-induced injury, we charted early systemic changes that could contribute to the development of vascular insulin resistance by measuring plasma biomarkers of inflammation and metabolic injury. Although we found that a 9-day CAP exposure did not affect blood glucose, plasma insulin levels, HOMA-IR or HOMA-β scores, or plasma lipids and proteins (Table 2), this short-term exposure did increase the abundance of plasma acrolein–protein adducts (Figure 3C) and plasma thiobarbituric acid reactive substances (TBARS) levels (Figure 3C, inset), which are reflective of increased systemic oxidative stress and lipid peroxidation (Uchida et al. 1998). These observations suggest that CAP exposure increases systemic oxidative stress and lipid peroxidation, changes that accompany vascular insulin resistance and inflammation (Figure 3A) and precede the development of overt systemic inflammation, systemic insulin resistance, and dyslipidemia (Table 2).

**Table 2:** Systemic effects of CAP exposure for 9 days (Study III).

Parameter, unit, *n*	Air	CAP	*p*	Air + HFD	CAP + HFD	*p*
Body weight, g, 14	27.43 ± 0.46	28.52 ± 0.61	0.163	29.36 ± 0.48	30.67 ± 0.40	0.046
Glucose, mg/dL, 14	160 ± 9	156 ± 7	0.700	167 ± 9	160 ± 6	0.489
Insulin, ng/mL, 4	0.46 ± 0.07	0.47 ± 0.06	0.898	0.85 ± 0.12	0.77 ± 0.13	0.653
HOMA-IR, 4	4.9 ± 1.0	4.5 ± 0.5	0.689	8.4 ± 0.5	8.5 ± 1.6	0.949
HOMA-β (%), 4	42.6 ± 4.8	48.4 ± 8.5	0.578	62.6 ± 6.0	73.1 ± 11.0	0.434
Adiponectin, ng/mL, 4	9.79 ± 1.90	7.02 ± 0.14	0.197	11.16 ± 2.62	13.17 ± 2.62	0.600
Leptin, ng/mL, 4	0.44 ± 0.05	0.50 ± 0.09	0.586	0.98 ± 0.33	0.84 ± 0.18	0.727
Resistin, ng/mL, 4	1.90 ± 0.87	1.92 ± 0.41	0.983	2.96 ± 1.20	2.33 ± 0.51	0.690
TNF-α, pg/mL, 4	4.53 ± 0.44	4.80 ± 1.33	0.851	4.11 ± 0.64	2.94 ± 0.47	0.212
IL-6, pg/mL, 4	8.41 ± 2.45	4.28 ± 1.25	0.184	8.45 ± 1.25	8.45 ± 1.13	0.999
TG, mg/dL, 9–10	33.56 ± 5.13	37.69 ± 4.96	0.571	16.80 ± 1.79	18.29 ± 2.51	0.634
Cholesterol, mg/dL, 9–10	66.07 ± 3.79	75.94 ± 6.55	0.222	110.41 ± 3.62	121.60 ± 6.19	0.136
HDL, mg/dL, 9–10	45.44 ± 1.96	50.81 ± 3.08	0.170	82.56 ± 2.51	93.70 ± 6.53	0.128
LDL, mg/dL, 9–10	10.38 ± 0.44	12.54 ± 1.11	0.100	18.62 ± 1.35	16.69 ± 0.61	0.210
HDL:LDL, 9–10	4.42 ± 0.21	4.16 ± 0.18	0.358	4.62 ± 0.31	5.78 ± 0.32	0.019
TP, g/dL, 9–10	4.32 ± 0.12	4.58 ± 0.15	0.198	4.33 ± 0.11	4.39 ± 0.08	0.670
Albumin, g/dL, 9–10	3.13 ± 0.07	3.25 ± 0.09	0.297	2.88 ± 0.06	2.93 ± 0.05	0.511
Albumin: TP, 9–10	0.73 ± 0.01	0.71 ± 0.01	0.242	0.67 ± 0.01	0.67 ± 0.01	0.818
ALT, U/L, 9–10	19.98 ± 1.00	26.36 ± 3.16	0.084	20.14 ± 1.94	17.59 ± 1.01	0.260
AST, U/L, 9–10	42.02 ± 4.11	51.08 ± 4.13	0.140	54.78 ± 2.96	53.99 ± 3.25	0.856
CK, U/L, 8–10	135.58 ± 16.99	132.64 ± 18.01	0.909	184.11 ± 29.64	183.60 ± 28.47	0.991
Notes: ALT, alanine aminotransferase; AST, aspartate aminotransferase; CAP, concentrated fine particulate matter; CK, creatinine kinase; HDL, high-density lipoprotein cholesterol; HOMA-β (= 20 × fasting plasma insulin levels [mU/L]/fasting blood glucose [mmol/L] – 3.5), %; HOMA-IR (= fasting blood glucose [mmol/L] × fasting plasma insulin levels [mU/L]/22.5); IL-6, interleukin 6; LDL, low-density lipoprotein cholesterol; TG, triglycerides; TNF-α, tumor necrosis factor α; TP, total protein. Blood and plasma parameters were measured in control or high-fat diet (HFD)-fed mice exposed for 30 days to air or CAP. Data represent the mean ± SE; *p*, air versus CAP.						

To determine whether this early oxidative stress was causally related to vascular insulin resistance and inflammation, we treated mice with the antioxidant TEMPOL and exposed them to air or to CAP (Figure 4, Study IV). Consistent with our previous results, we found that CAP exposure attenuated insulin-stimulated Akt phosphorylation and decreased IκBα levels in the aorta; however, TEMPOL treatment prevented both changes (Figure 4A,B). These results support the notion that CAP exposure induces oxidative stress that, in turn, suppresses vascular insulin signaling and induces vascular inflammation.

### Role of Pulmonary Oxidative Stress in Mediating CAP-Induced Vascular Insulin Resistance and Inflammation

Given that the lungs are the first and major site of inhaled particle deposition and toxicity (Oberdörster et al. 2005), we measured oxidative stress in the lungs of CAP-exposed mice to determine whether CAP exposure induced pulmonary oxidative stress. We measured changes in pulmonary antioxidant genes because an increase in the expression of these genes would be reflective of pulmonary oxidative stress. We found that 9-day CAP exposure caused an increase in the expression of the antioxidant genes SOD2, SOD3, and GST-α (see Table S6) and a corresponding increase in abundance of the proteins ecSOD (SOD3, extracellular superoxide dismutase, Figure 5A) in the lungs, which could be indicative of pulmonary oxidative stress. However, indices of pulmonary inflammation (see Table S3) were unaffected, suggesting that CAP exposure results in acute pulmonary oxidative stress without triggering overt lung inflammation.

The increase in the levels of antioxidant enzymes in the lungs of CAP-exposed mice led us to ask whether oxidative stress in the lungs was related to the development of vascular insulin resistance and inflammation in CAP-exposed mice. Therefore, we tested whether increasing the antioxidant capacity of the lung would diminish these vascular effects of CAP. To enhance the antioxidant capacity of the lung, we exposed mice transgenic for lung-specific ecSOD (ecSOD-Tg) (Folz et al. 1999), which show lung-restricted overexpression of the transgenic ecSOD gene (Figure 5B), to air or CAP for 9 days (Study V) and examined changes in protein–acrolein adducts in the lungs. As shown in Figure 5C, CAP exposure increased the levels of protein–acrolein adducts in the lungs of WT mice (Figure 5Ci) but not in those of ecSOD-Tg mice (Figure 5Cii), indicating that overexpression of ecSOD prevented CAP-induced pulmonary oxidative stress. Remarkably, we found that the overexpression of ecSOD exclusively in the lung also prevented CAP-induced oxidative stress in peripheral tissues such as circulating lymphocytes and the aorta (see Figure S3A,B). Moreover, as shown in Figure 5D,E, even though CAP exposure attenuated insulin sensitivity and decreased IκBα levels in the aortas of WT mice, these effects were absent in CAP-exposed ecSOD-Tg mice. Similarly, overexpression of pulmonary ecSOD also attenuated the CAP-induced decrease in plasma NO_x_ levels (see Table S7). Taken together, these results suggest that the overexpression of ecSOD in the lungs prevents the development of vascular insulin resistance and inflammation in CAP-exposed mice. These results also support the idea that CAP exposure increases pulmonary oxidative stress, which in turn triggers systemic oxidative stress, leading to the development of vascular insulin resistance and inflammation.

## Discussion

This study shows that short-term exposure to PM_2.5_ decreased diet-independent vascular insulin sensitivity, which was mediated in part by oxidative stress in the lungs. These findings provide a novel link between pulmonary oxidative stress and vascular insulin signaling by showing that pulmonary oxidative stress is sufficient to induce insulin resistance and inflammation in blood vessels of mice exposed to PM_2.5_. Because insulin resistance localized to the vasculature is a subclinical effect that could occur in the absence of measurable systemic effects, this outcome has not been observed in previous clinical studies. Nevertheless, vascular insulin resistance might represent a critical first step of the mechanism by which PM_2.5_ exposure accelerates and exacerbates the risk for both cardiovascular and metabolic diseases in humans.

Previous studies have shown that acute and chronic exposures to ambient levels of PM_2.5_ are associated with an increase in the prevalence of CVD and diabetes as well as enhanced systemic insulin resistance and diabetes-related mortality in humans (Brook et al. 2010, 2013a; Chen et al. 2013; Coogan et al. 2012; Eze et al. 2015; Park et al. 2015; Pearson et al. 2010; Pope et al. 2015; Rao et al. 2015). Nevertheless, the mechanisms underlying these changes remain unclear. The ability of PM_2.5_ to decrease systemic insulin sensitivity has also been demonstrated in animal models, which show that prolonged (6–10 months) PM_2.5_ exposure increases systemic insulin resistance (Sun et al. 2009; Xu et al. 2011). However, our observation that short-term PM_2.5_ exposure induces vascular insulin resistance without affecting systemic insulin sensitivity suggests that in comparison with other tissues, the blood vessels are more sensitive to PM_2.5_ exposure and that vascular insulin resistance could be a contributing factor to the development of CVD and diabetes in humans exposed to ambient air pollution. In particular, the observed decrease in insulin-stimulated eNOS phosphorylation in CAP-exposed animals suggests that PM_2.5_-induced endothelial insulin resistance could be a key event in the mechanism, triggering the onset of other deleterious cardiovascular outcomes and systemic insulin resistance after PM_2.5_ exposure. Phosphorylation of eNOS by insulin increases eNOS activity and NO production, which regulates vascular tone, thrombosis, and atherogenesis (Kim et al. 2008; Kim et al. 2006; Landmesser et al. 2004). Therefore, suppression of insulin-stimulated phosphorylation of eNOS could account for many of the vascular effects of PM_2.5_ such as the increases in blood coagulation, blood pressure, vascular dysfunction, and atherogenesis (Brook et al. 2010). Previous work has shown that cardiovascular deaths account for the majority of premature mortality associated with exposure to particulate air pollution, and these outcomes have been linked to an increase in inflammation as well as to PM-induced dysfunction in the endothelium and in the autonomic and central nervous systems (Brook et al. 2010). Although changes in each of these processes could individually elevate CVD risk, it remains unclear whether PM_2.5_ provokes cardiovascular and metabolic injury by affecting all of these processes simultaneously or by affecting only a core set of “sensitive” processes. The present data, showing that exposure to PM_2.5_ suppresses vascular insulin signaling, suggest that vascular insulin resistance could be one such “sensitive” target of PM_2.5_ that in turn affects many different processes such as tissue perfusion, endothelial function, and atherogenesis (Kim et al. 2006; Kubota et al. 2011; Landmesser et al. 2004). Collectively, theses changes could increase cardiometabolic risk and cardiovascular mortality in humans exposed to PM_2.5_. In this regard, it is significant to note that the effects observed in our study occurred at exposure levels near the National Ambient Air Quality Standard for PM_2.5_ (12 μg/m^3^) [Environmental Protection Agency (EPA) 2016] and are within the range of human exposure to daily PM_2.5_ concentrations commonly found (5–50 μg/m^3^) in U.S. cities, yet far below PM_2.5_ levels (> 100 μg/m^3^) regularly observed in India and China (Brook et al. 2010). The sensitivity of mice to PM_2.5_ exposure is consistent with data from human studies showing that an acute increase in PM_2.5_ of only 1.5 μg/m^3^ (9.7 ± 3.9 to 11.2 ± 3.9 μg/m^3^) enhances HOMA-IR (Brook et al. 2013b), and an increase of 10 μg/m^3^ enhances the diabetes prevalence by 1% in chronically exposed individuals living in the southeast United States (Pearson et al. 2010).

Previous work with rodent models of diet-induced obesity has shown that early vascular insulin resistance could be a critical contributing factor to the subsequent development of systemic glucose intolerance and frank diabetes (Kim et al. 2008; Kubota et al. 2011). In these models, an early decrease in insulin sensitivity in the aorta (after 1–2 weeks feeding an HFD) is followed by the development of insulin resistance in the skeletal muscle and liver (8 weeks) and then in the adipose tissue (14 weeks) (Kim et al. 2008). Moreover, vascular insulin resistance caused by the deletion of endothelial-specific insulin receptor substrate 2 (IRS-2) has been found to be sufficient for the development of systemic glucose intolerance (Kubota et al. 2011). In light of these data, we suggest that as in the development of diet-induced insulin resistance, the development of PM_2.5_-induced insulin resistance also starts in the vasculature and then extends to other peripheral organs and tissues such as skeletal muscle, liver and adipose tissue. Nevertheless, we cannot exclude the possibility that changes in the liver, skeletal muscle, or adipose tissue also contribute to the systemic effects of PM_2.5_. Chronic exposure to PM induces hepatic endoplasmic reticulum (ER) stress (Laing et al. 2010), a process implicated in the development of insulin resistance and diabetes (Hotamisligil 2010), which could potentially contribute to the development of systemic insulin resistance induced by CAP exposure, particularly in the setting of diet-induced obesity. Similarly, inflammation in adipose tissue could be a contributing factor. Indeed, we found that macrophage infiltration in the adipose tissue of HFD-fed mice was increased by CAP exposure (see Figure S1D), which could also contribute to the development of systemic insulin resistance; previous studies have shown that diet-induced obesity is associated with macrophage infiltration in the adipose tissue and that depletion of macrophages prevents systemic insulin resistance (Bu et al. 2013). We found that although CAP exposure enhanced the mRNA levels of TNF-α and MIP-1α in the adipose tissue of HFD-fed mice, presumably because of an increase in infiltrating macrophages, it reduced HFD-induced increases in adipose tissue leptin mRNA levels and adipocyte hypertrophy (see Figure S1C–E). The mechanisms underlying these effects of PM_2.5_ and their contributions to the development of systemic insulin resistance remain unclear, but excessive TNF-α production in the adipose tissue could induce lipolysis (Suganami and Ogawa 2010) and thereby decrease adipocyte size.

Although the mechanisms by which HFD induces insulin resistance remain unclear, it has been suggested that oxidative stress plays a critical role in this process (Paneni et al. 2015). Therefore, at least in principle, an increase in ROS production in PM_2.5_-exposed mice (and humans) could be one mechanism by which PM_2.5_ exposure increases insulin resistance, as was also suggested by a previous study with p47(phox)-deficient mice (Xu et al. 2010). A critical role of ROS in mediating the effects of PM_2.5_ is supported by our observations that CAP exposure increased the levels of antioxidant enzymes, such as ecSOD, in the lungs and that PM_2.5_-induced vascular insulin resistance was prevented by TEMPOL, which catalyzes the disproportionation of superoxide (Krishna et al. 1996). Even though it remains unclear whether the increased sensitivity of HFD mice to PM_2.5_ is a result of excessive perturbations in tissue redox signaling and inadequate antioxidant response, the present data indicate that vascular insulin resistance in PM_2.5_-exposed mice may be attributable, at least in part, to oxidative stress induced by PM_2.5_ exposure. PM_2.5_ contains several prooxidant molecules, particularly metals such as iron, zinc, nickel, and chromium, that can trigger the formation of superoxide and other related ROS, as well as polycyclic aromatic hydrocarbons and quinones that can undergo redox cycling to generate ROS (Ghio et al. 2012). Indeed, compositional analysis of the Louisville CAP from our exposure studies showed high levels of iron (8–12%, see Table S2). Consistent with a central role of ROS in mediating PM toxicity, it has been shown that the toxicity of airborne particles correlates with their ability to generate ROS and that exposure to such particles induces oxidative stress in different tissues and cell lines (Ghio et al. 2012).

If the vascular effects of PM_2.5_ are mediated by oxidative stress, then where is this stress initiated, and how does it affect vascular insulin signaling? It has been suggested that systemic oxidative stress is due to either PM_2.5_ particles deposited in the lung that induce pulmonary oxidative stress, which then spreads systemically, or that particles or particle constituents leak from the lung into the systemic circulation and then cause oxidative injury in peripheral tissues (Brook et al. 2010). Our finding that overexpression of ecSOD only in the lung prevented vascular insulin resistance suggests that pulmonary oxidative stress *per se* is sufficient to cause PM_2.5_-induced vascular insulin resistance and that vascular insulin resistance cannot be attributed to a diffusion of particles or particle constituents from the lung into the circulation but rather is an indirect consequence of pulmonary oxidative stress. Nevertheless, how oxidative stress is transmitted from the lung to the blood vessels remains unclear. Kampfrath et al. (2011) suggested that pulmonary oxidative stress generates oxidized lipids [e.g., 1-palmitoyl-2-(5-oxovaleroyl)-sn-glycero-3-phosphocholine (POVPC)] that diffuse from the lung to peripheral tissues, triggering tissue-specific injury. However, diffusible lipids that arise in the lung and interfere with vascular insulin signaling are yet to be identified unambiguously, and further experiments are required to elucidate their role in inducing vascular insulin resistance secondary to pulmonary oxidative stress. Nevertheless, our observations here show that pulmonary oxidative stress *per se* plays a central role in the peripheral effects of PM_2.5_ and suggest a fundamental, but poorly understood, link between pulmonary oxidative stress and vascular insulin signaling. Like PM_2.5_ exposure, other insults that cause pulmonary oxidative stress, such as exposure to tobacco smoke or ozone, have also been associated with the development of systemic insulin resistance (Bass et al. 2013; Henkin et al. 1999; Vella et al. 2015). Similarly, pulmonary diseases such as microbial infections (Wang et al. 2009) and asthma (Gulcan et al. 2009) are also associated with insulin resistance and an increased risk for diabetes. Although it remains to be seen whether an increase in pulmonary oxidative stress induced by various toxicological or pathological insults is a general cause of insulin resistance and diabetes, it is tempting to speculate that the vascular endothelium is particularly sensitive to oxidants generated in the lung and that there is an underappreciated link between pulmonary oxidative stress and the onset and precipitation of metabolic disease.

## Conclusions

We found that upon exposure to PM_2.5_, mice developed vascular insulin resistance associated with vascular inflammation and dysfunction. These changes occurred even in the absence of an HFD but were similar to vascular changes observed in models of diet-induced obesity. The results of our experiments with mice treated with the antioxidant TEMPOL and with mice overexpressing ecSOD in the lung suggest that vascular insulin resistance is secondary to oxidative stress in the lung. On the basis of these observations, we suggest a model in which PM_2.5_ deposited in the lung generates ROS, which in turn generate a diffusible mediator(s) that interferes with vascular insulin signaling by attenuating insulin-stimulated Akt and eNOS phosphorylation in blood vessels (see Figure S5). Further studies are required to identify the mediators of this process and to determine how secondary oxidative products arising in the lung cause vascular insulin resistance. Nevertheless, our observations suggest that the cardiovascular, and possibly the metabolic, effects of PM_2.5_ could be mitigated by improving lung health or by targeting antioxidant interventions to the lung. Increasing the antioxidant capacity of the lung is likely not only to delay the progression of chronic cardiopulmonary injury caused by chronic PM_2.5_ exposure but might also mitigate its acute cardiovascular effects. Conversely, conditions associated with a decrease in the antioxidant capacity of the lung, such as smoking, asthma, advanced age, or influenza, could increase the susceptibility of affected individuals to the cardiovascular effects of PM. Indeed, some studies have suggested that smokers (Miller et al. 2007; Pope et al. 2004), individuals of advanced age (Andersen et al. 2008; Samoli et al. 2008), and individuals with asthma (Yeatts et al. 2007) or influenza (Chen et al. 2011) are much more susceptible to the cardiovascular effects of PM exposure, but more work is needed to establish whether lung health is a risk factor for these effects. Our model provides a new template for understanding how PM_2.5_ exposure, by inducing vascular insulin resistance, could simultaneously affect disparate cardiovascular processes such as thrombosis, autonomic dysfunction, blood pressure regulation, and atherogenesis as well as metabolic changes critical for the development of diabetes.

## Supplemental Material

(1.3 MB) PDF
