# Supplementary material for: Exposure to Fine Particulate Air Pollution Causes Vascular Insulin Resistance by Inducing Pulmonary Oxidative Stress
Source: Environ Health Perspect. 2016 Apr 29;124(12):1830–9. doi: 10.1289/EHP212 (PMC5132639; doi:10.1289/EHP212)
Supplement: (1.3 MB) PDF [file EHP212.s001.acco.pdf]

Environ Health Perspect
DOI: 10.1289/EHP212

Note to readers with disabilities: *EHP* strives to ensure that all journal content is accessible to all readers. However, some figures and Supplemental Material published in *EHP* articles may not conform to 508 standards due to the complexity of the information being presented. If you need assistance accessing journal content, please contact ehp508@niehs.nih.gov. Our staff will work with you to assess and meet your accessibility needs within 3 working days.

Supplemental Material

Exposure to Fine Particulate Air Pollution Causes Vascular Insulin Resistance by Inducing Pulmonary Oxidative Stress

Petra Haberzettl, Timothy E. O'Toole, Aruni Bhatnagar, and Daniel J. Conklin

Table of Contents

Methods

Figure S1: Effects of CAP exposure on adiposity and adipose inflammation. (**A**) Changes in body weight during the 30-day exposure to air or CAP in mice fed control (13% kcal fat) or high fat diet (HFD, 60% kcal fat**,** Study I). Data are mean ± SE (* $p < 0.05$ HFD vs. matching controls; $^{\#}$ $p < 0.05$ air vs. CAP; n = 8). **(B)** Epididymal adipose tissue mass and quantification of (C) adipocyte size and (D) cells positive for F4/80 (F4/80^{+}-cells) and crown-like structures (CLS) in epididymal adipose tissue of control (10% kcal fat) or HFD-fed mice exposed for 30 days to air or CAP (Study II). Epididymal adipose tissue sections were labeled with F4/80 and stained with H&E or fluorescence-labeled with Texas red and DAPI. (E) Adipose tissue mRNA levels of tumor necrosis factor-α (TNF-α), macrophage inflammatory protein-1α (MIP-1α), monocyte chemotactic protein-1 (MCP-1), interleukin-6 (IL-6), interleukin-1β (IL-1β), leptin, adiponectin and peroxisome proliferators activated receptor γ (PPARγ) in control diet or HFD-fed mice exposed for 30 days to air or CAP (Study II). Data are mean ± SE (* $p < 0.05$ HFD vs. matching controls; $^{\#}$ $p < 0.05$ air vs. CAP; n = 4).

Figure S2: Cardiovascular effects of CAP exposure. **(A)** Western blot analysis of insulin stimulated Akt phosphorylation in hearts (n = 5-7) isolated from control (13% kcal fat) or high fat diet (HFD, 60% kcal fat) fed mice exposed for 9 days to air or CAP (Study III). Insulin-stimulated Akt phosphorylation was examined *ex vivo* in hearts perfused in the Langendorff mode (Wetzelberger et al. 2010) with saline or insulin (150 mU) for 10min. **(B)** Western blot analysis of insulin-stimulated ERK (p44/p42) phosphorylation (n = 10) in aortas isolated from control diet fed mice exposed for 9 days to air or CAP. Data are mean ± SE (* $p < 0.05$ control vs. insulin; $^{\#}$ $p < 0.05$ vs. insulin stimulated air-exposed control group). **(C)** Aortic contractility in response to phenylephrine (PE) and high potassium (HI K^+) or thromboxane A_2 analog (U46,619) in control or HFD-fed mice exposed for 9 (i, n = 4) or 30 (ii, n = 6-8) days to air or CAP (Study I and III). Data are mean ± SE (* $p < 0.05$, $^{+}$ $0.10 > p < 0.05$ vs. air-exposed control group).

Figure S3: CAP-induced oxidative stress in lymphocytes and aorta is prevented in lung-specific ecSOD transgene (ecSOD-Tg) mice. **(A)** Flow cytometry analysis of the monochlorobimane (MCB) fluorescence in blood lymphocytes of (i) WT and (ii) ecSOD-Tg mice exposed for 9 days to air or CAP (Study V). Representative flow cytometry plots of side (SSC) and forward (FSC) scatter (left) used to define the lymphocyte cell population for the analysis of MBC-fluorescence. Representative histograms and quantification (right) of the median MBC fluorescence in the gated lymphocytes demonstrate a CAP-induced decrease in cellular GSH in WT but not in ecSOD-Tg mice. Data are mean ± SE ($^{\#}$ $p < 0.05$, air vs. CAP; n = 8-12). **(B)** Representative Western blots and densitometric analysis of the abundance protein-acrolein adducts in the aorta isolated from WT (i) and ecSOD-Tg (ii) mice exposed for 9 days to air or CAP. Data are mean ± SE ($^{\#}$ $p < 0.05$, $^{+}$ $p < 0.1$ air vs. CAP; n = 8).

Figure S4: Full width IκBα Western blots shown in Figures 3Aiv and 4B and protein-acrolein adduct Western blots including loading controls (protein, amido black protein stain) shown in Fig. 3C, Fig. 5C and Supplemental Material, Fig. S3B.

Figure S5: Proposed mechanism by which the exposure to $PM_{2.5}$ increases the risk for the development of both CVD and T2D. Inhaled $PM_{2.5}$ particles deposited in the lungs generate reactive oxygen species (ROS, e.g. the formation of superoxide, $O_2^{\cdot-}$) and lipid peroxidation products. The induction of pulmonary oxidative stress leads to the generation of a diffusible mediator(s) that transfers the oxidative stress response into peripheral tissues (e.g. blood

lymphocytes and aorta). This triggers the development of inflammation and insulin resistance in blood vessels. The induction of vascular insulin resistance in turn affects cardiovascular processes such as endothelium dysfunction, thrombosis, blood pressure regulation, tissue perfusion and atherogenesis that promote the development of both CVD and diabetes. Treatment with the antioxidant TEMPOL and lung-specific overexpression of ecSOD leads to catalyzed disproportionation of superoxide and prevents $PM_{2.5}$-induced vascular insulin resistance and inflammation, suggesting that vascular insulin resistance and inflammation are secondary to oxidative stress particularly in the lung.

Table S1: RT-PCR primer.

Table S2: CAP exposure concentrations, enrichment factors, sizes and elemental compositions of the exposures in Study I-V.

Table S3: Effects of CAP exposure for 30 or 9 days on lung inflammation (Study I, III).

Table S4: Mean CAP concentrations (determined by filter weight) of exposures performed from July 2010 to August 2013 (see Fig. 3B).

Table S5: Vascular effects of 9 or 30 days CAP exposure (Study I and III).

Table S6: Effects of CAP exposure for 9 days on lung antioxidant defense.

Table S7: Systemic effects of CAP exposure for 9 days in WT and ecSOD-Tg mice (Study V).

References

METHODS

Animals. Male mice (≈12-weeks old) were exposed to HEPA-filtered air or concentrated ambient $PM_{2.5}$ (CAP, 6 h/d) consecutively for 9 or 30 days as described (Haberzettl et al. 2012; O'Toole et al. 2010). Mice were exposed to CAP concentrations varying between 30-120 μg/m^3 with similar mean diameter and elemental particle composition for the different exposures (see Supplemental Material, Table S2). The versatile aerosol concentration enrichment system (VACES) concentrates $PM_{2.5}$ form outdoor air without changing their chemical composition or physical properties (Haberzettl et al. 2012) up to 10 fold resulting in exposure concentration of 30-120 μg/m^3. This corresponds to an exposure of 7.5 to 30 μg/m^3 in 24h, and is comparable to both the CAP concentrations used in previous animal studies (Sun et al. 2009; Xu et al. 2011) and the 24h average levels of $PM_{2.5}$ in most major US cities that vary from 20-35 μg/m^3 (Brook et al. 2010). Male C57BL/6J mice (Jackson Laboratory) were either fed continuously with a control diet containing 13% kcal fat (LabDiets, Study I and III) or were placed at 8-weeks of age on a diet containing 10% kcal fat (Research Diets; Study II) or a high-fat diet (HFD, 60% kcal fat, Research Diets, Study I, II and III). After 4 weeks (12-weeks of age) mice were exposed for 9 (Study III, 14 mice per group) and 30 (Study I, II, 8 mice per group) days to air or CAP while continuing the respective diet. Because treatment with the superoxide dismutase mimetic TEMPOL (4-hydroxy-2,2,6,6-tetramethylpiperidine1-oxyl, 1 mM in drinking water) has been shown to improve vascular endpoints under oxidative stress conditions such as diabetes and exposure to air pollution (Lund et al. 2009; Yadav and Harris 2011) another group of C57BL/6 mice was treated with 1 mM TEMPOL (Sigma-Aldrich, daily dose of approximately ~25.5 mg/kg/day, daily fresh prepared) in drinking water. Mice treated with TEMPOL for 2 days were then exposed for 9 days to air or CAP continuing the TEMPOL treatment (Study IV, 5-10 mice

per group). To specifically attenuate pulmonary oxidative stress, mice transgenic (Tg) for lung-specific extracellular superoxide dismutase (ecSOD, Folz et al. 1999) were exposed for 9 days to air or CAP (Study V).

Immunoblotting. Western blot analysis was performed as described (Haberzettl et al. 2012; Wheat et al. 2011) using antibodies against phospho-Akt (Ser473), Akt, phospho-eNOS (Ser1177), eNOS, phospho-ERK (Thr202/204), ERK (p44/42), IκBα, β-Tubulin (1:1000; Cell Signaling Technology), actin (1:10,000, Sigma-Aldrich), ecSOD (1:1000; Enzo Life Science), transgene (*t)*-ecSOD or protein-acrolein adducts.

Real Time-PCR. RNA was isolated from visceral adipose tissue and lungs using the RNeasy Lipid Tissue Mini kit (Qiagen) or the Exiqon miRCURY^{TM} RNA isolation kit (Exiqon), respectively. Quantitative real-time PCR (RT-PCR) was performed as described (Haberzettl et al. 2009), using the primer listed in Supplemental Material, Table S1 and an IL-6 primer purchased from SA Bioscience (Qiagen).

Immunohistochemistry. Formalin-fixed, paraffin-embedded tissue sections (4 μm) of visceral adipose tissue (epididymal) were labeled with F4/80 (1:1500; Novocastra) used with either a Vector Elite kit (DAB; Dako) or a Texas red anti-mouse IgG (1:500) and *SlowFade*® Gold antifade (Molecular Probes, Invitrogen). Adipocyte size, F4/80 positive cells (F4/80^{+}-cells) and crown-like structures (CLS) were quantified in 10 random microscopic fields of 4 specimens (Hellmann et al. 2011).

Vascular reactivity. Thoracic aortas were isolated and vascular reactivity was assayed as described (Conklin et al. 2009). Briefly, endothelium-dependent relaxation was measured in phenylephrine-precontracted (PE; 10 μM) aortas relaxed with acetylcholine (ACh; 0.1 nM-10

μM). Endothelium-independent relaxation was measured in aortas precontracted with either 100 mM potassium (Study I and III) or with U46,619 (thromboxane A_2 mimetic, 0.1 μM; Study III) relaxed with sodium nitroprusside (SNP; 0.1 nM-10 μM). Relaxation was calculated as a percentage reduction of agonist-induced tension. Tension was normalized to each aortic ring's cross-sectional area (mN/mm^2).

Flow cytometry. To examine systemic oxidative stress by flow cytometry, cellular GSH-levels were analyzed by measuring monochlorobimane (MCB) fluorescence intensity (Haberzettl et al. 2014; Sithu et al. 2010) in circulating cells. For this, collected blood was lysed (10 min, RT, BD PharmLyse, BD BioSciences) and cells were incubated for 15min with MCB (20 μM, RT; Sigma-Aldrich). The median MCB fluorescence was detected in the circulating lymphocyte population gated based on side (SSC) and forward scatter (FSC, see Supplemental Material, Fig. S3A) using an LSRII flow cytometer (BD BioSciences) and the FlowJo version 8 software (Treestar software).

Supplemental Material, Figure S1

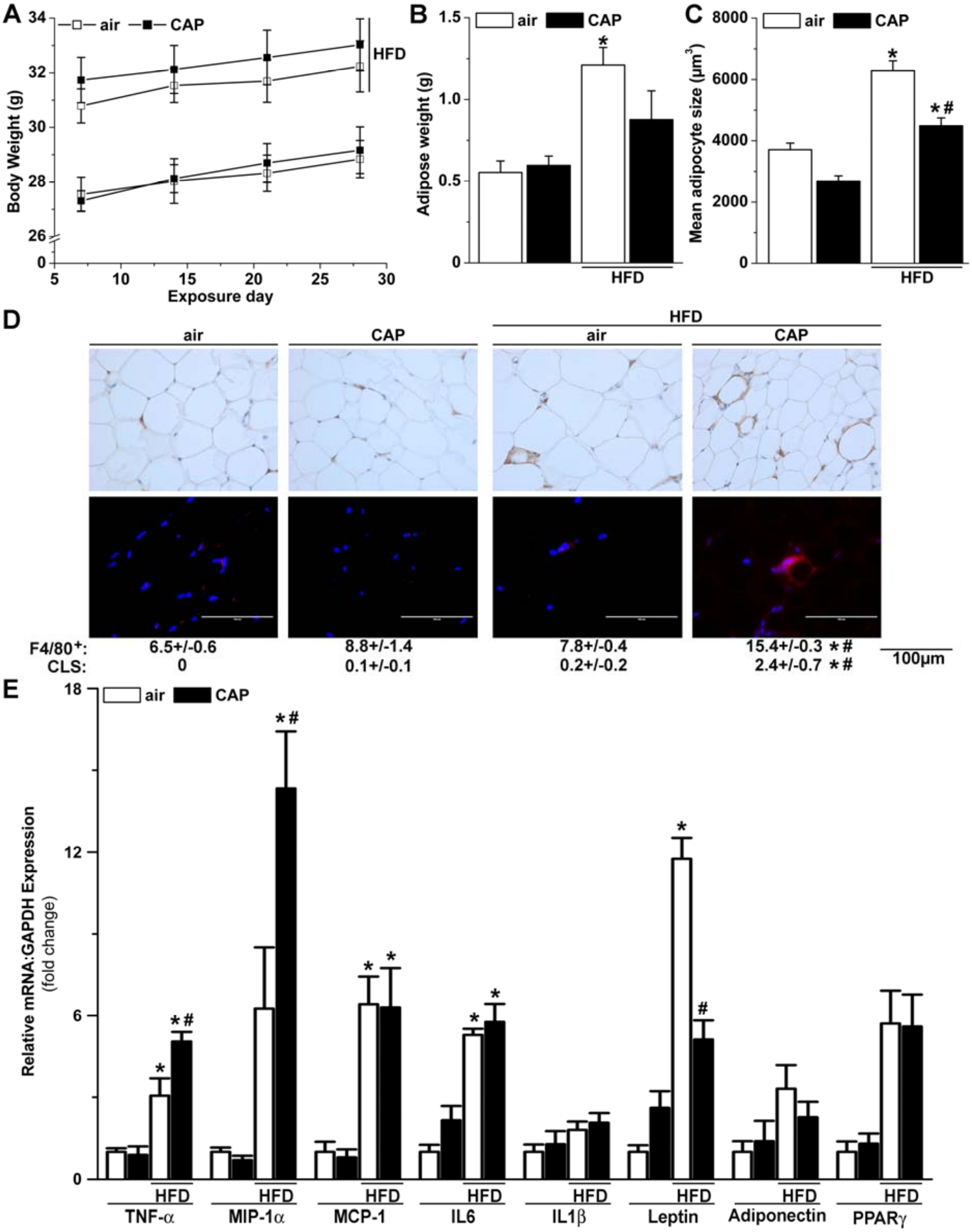

Figure S1: Effects of CAP exposure on adiposity and adipose inflammation. (**A**) Changes in body weight during the 30-day exposure to air or CAP in mice fed control (13% kcal fat) or high fat diet (HFD, 60% kcal fat**,** Study I). Data are mean ± SE (* $p < 0.05$ HFD vs. matching controls; $^{\#}$ $p < 0.05$ air vs. CAP; n = 8). **(B)** Epididymal adipose tissue mass and quantification of (C) adipocyte size and (D) cells positive for F4/80 (F4/80^{+}-cells) and crown-like structures (CLS) in epididymal adipose tissue of control (10% kcal fat) or HFD-fed mice exposed for 30 days to air or CAP (Study II). Epididymal adipose tissue sections were labeled with F4/80 and stained with H&E or fluorescence-labeled with Texas red and DAPI. (E) Adipose tissue mRNA levels of tumor necrosis factor-α (TNF-α), macrophage inflammatory protein-1α (MIP-1α), monocyte chemotactic protein-1 (MCP-1), interleukin-6 (IL-6), interleukin-1β (IL-1β), leptin, adiponectin and peroxisome proliferators activated receptor γ (PPARγ) in control diet or HFD-fed mice exposed for 30 days to air or CAP (Study II). Data are mean ± SE (* $p < 0.05$ HFD vs. matching controls; $^{\#}$ $p < 0.05$ air vs. CAP; n = 4).

Supplemental Material, Figure S2

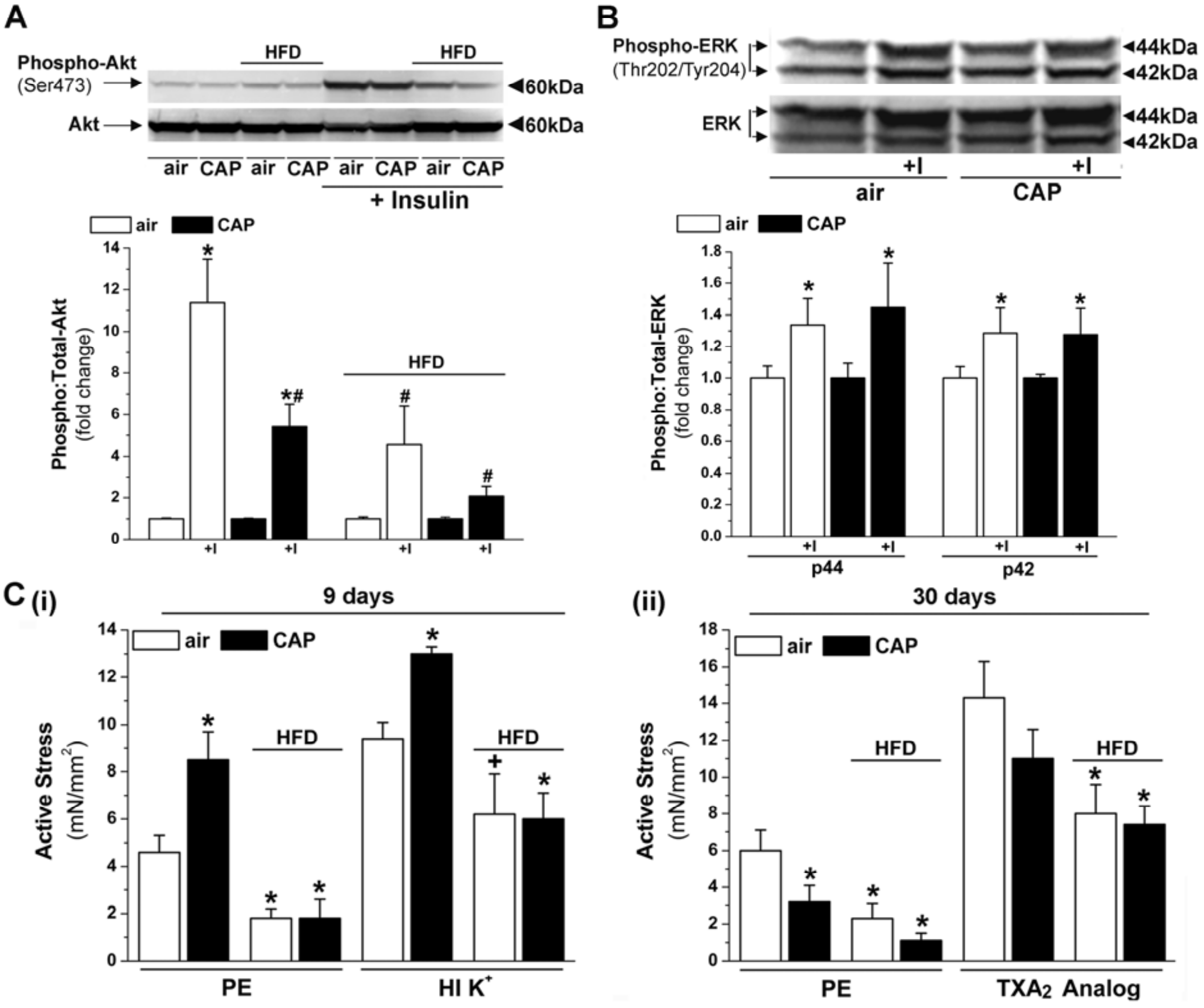

Figure S2: Cardiovascular effects of CAP exposure. **(A)** Western blot analysis of insulin stimulated Akt phosphorylation in hearts (n = 5-7) isolated from control (13% kcal fat) or high fat diet (HFD, 60% kcal fat) fed mice exposed for 9 days to air or CAP (Study III). Insulin-stimulated Akt phosphorylation was examined *ex vivo* in hearts perfused in the Langendorff mode (Wetzelberger et al. 2010) with saline or insulin (150 mU) for 10min. **(B)** Western blot analysis of insulin-stimulated ERK (p44/p42) phosphorylation (n = 10) in aortas isolated from control diet fed mice exposed for 9 days to air or CAP. Data are mean ± SE (* $p < 0.05$ control vs. insulin; $^{\#}$ $p < 0.05$ vs. insulin stimulated air-exposed control group). **(C)** Aortic contractility

in response to phenylephrine (PE) and high potassium (HI K^+) or thromboxane A_2 analog (U46,619) in control or HFD-fed mice exposed for 9 (i, n = 4) or 30 (ii, n = 6-8) days to air or CAP (Study I and III). Data are mean ± SE (* $p < 0.05$, $^+$ $0.10 > p < 0.05$ vs. air-exposed control group).

Supplemental Materail, Figure S3

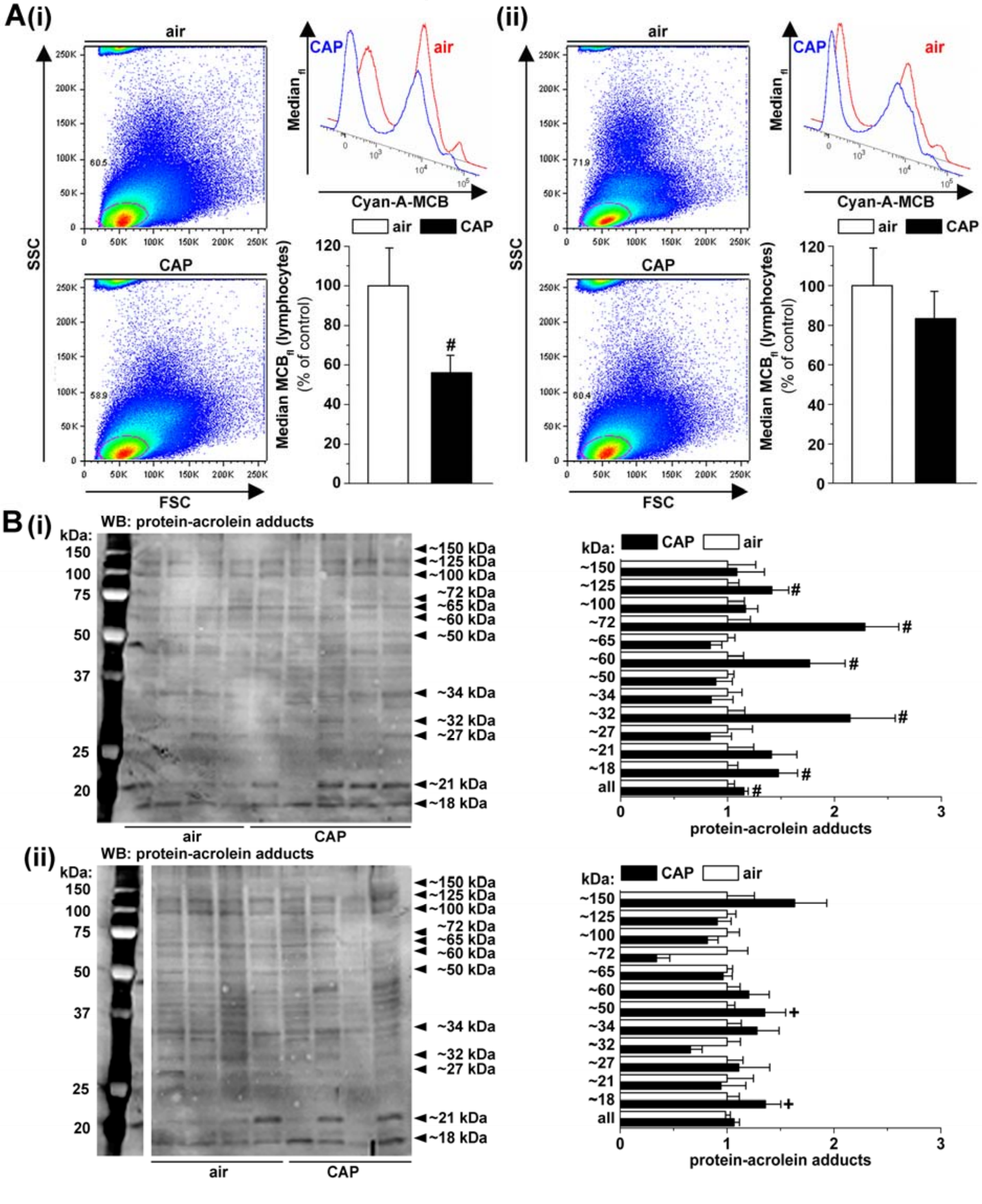

Figure S3: CAP-induced oxidative stress in lymphocytes and aorta is prevented in lung-specific ecSOD transgene (ecSOD-Tg) mice. (A) Flow cytometry analysis of the

monochlorobimane (MCB) fluorescence in blood lymphocytes of (i) WT and (ii) ecSOD-Tg mice exposed for 9 days to air or CAP (Study V). Representative flow cytometry plots of side (SSC) and forward (FSC) scatter (left) used to define the lymphocyte cell population for the analysis of MBC-fluorescence. Representative histograms and quantification (right) of the median MBC fluorescence in the gated lymphocytes demonstrate a CAP-induced decrease in cellular GSH in WT but not in ecSOD-Tg mice. Data are mean ± SE ($^{\#}$ $p < 0.05$, air vs. CAP; n = 8-12). **(B)** Representative Western blots and densitometric analysis of the abundance protein-acrolein adducts in the aorta isolated from WT (i) and ecSOD-Tg (ii) mice exposed for 9 days to air or CAP. Data are mean ± SE ($^{\#}$ $p < 0.05$, $^{+}$ $p < 0.1$ air vs. CAP; n = 8).

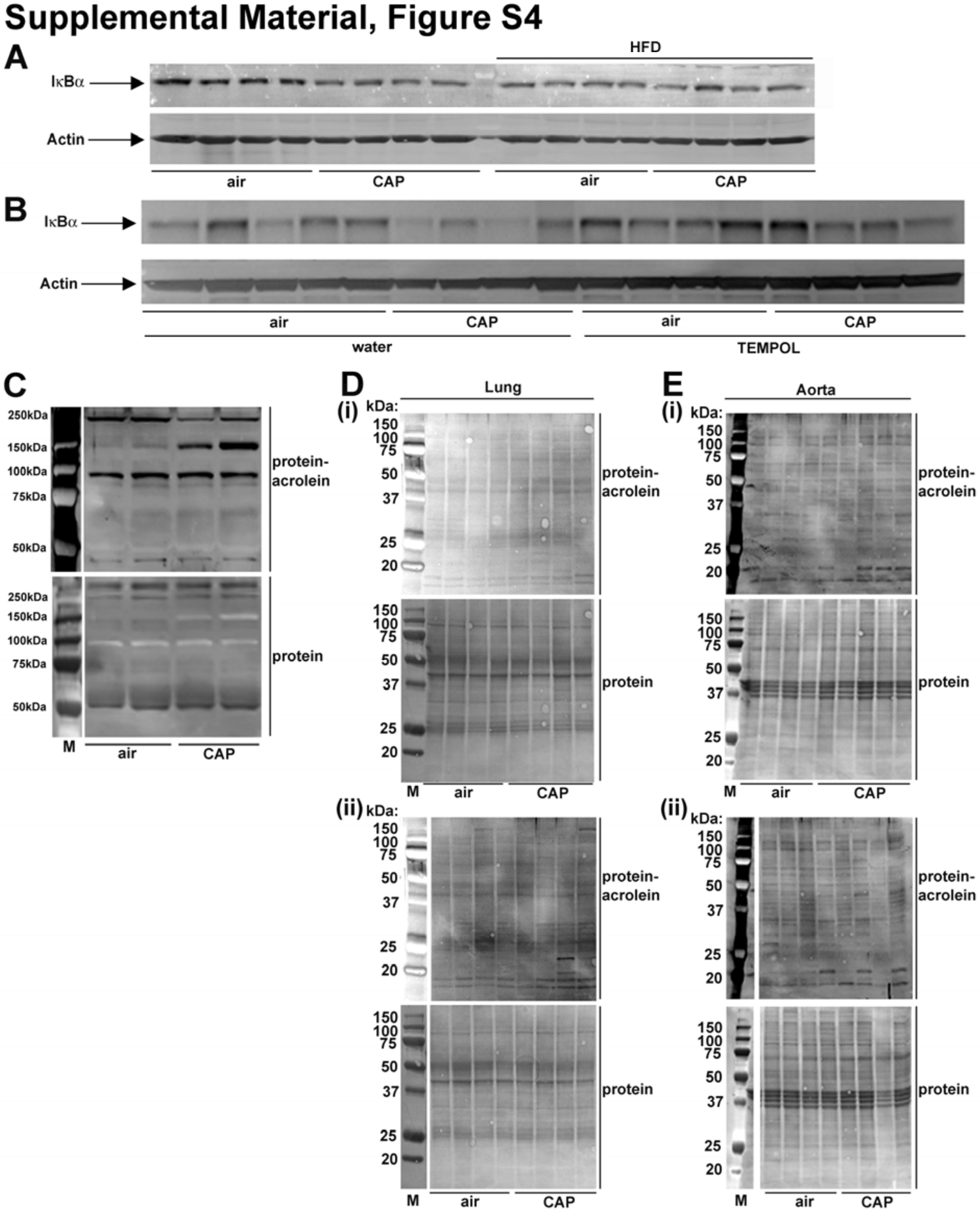

Figure S4: Full width IκBα Western blots shown in Figures 3Aiv and 4B and protein-acrolein adduct Western blots including loading controls (protein, amido black protein stain) shown in Fig. 3C, Fig. 5C and Supplemental Material, Fig. S3B.

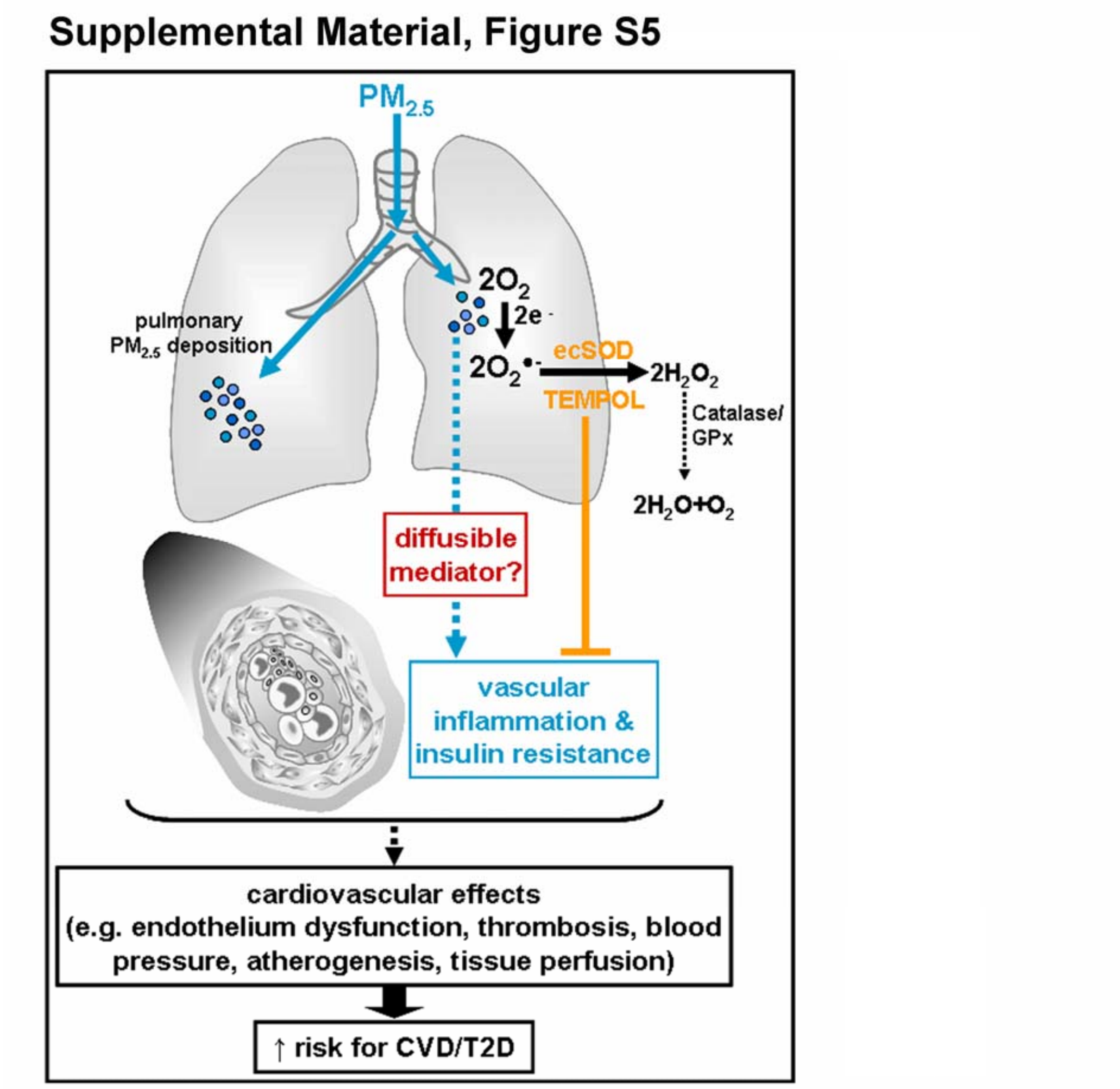

Figure S5: Proposed mechanism by which the exposure to $PM_{2.5}$ increases the risk for the development of both CVD and T2D. Inhaled $PM_{2.5}$ particles deposited in the lungs generate reactive oxygen species (ROS, e.g. the formation of superoxide, $O_2^{\cdot-}$) and lipid peroxidation products. The induction of pulmonary oxidative stress leads to the generation of a diffusible mediator(s) that transfers the oxidative stress response into peripheral tissues (e.g. blood lymphocytes and aorta). This triggers the development of inflammation and insulin resistance in blood vessels. The induction of vascular insulin resistance in turn affects cardiovascular

processes such as endothelium dysfunction, thrombosis, blood pressure regulation, tissue perfusion and atherogenesis that promote the development of both CVD and diabetes. Treatment with the antioxidant TEMPOL and lung-specific overexpression of ecSOD leads to catalyzed disproportionation of superoxide and prevents $PM_{2.5}$-induced vascular insulin resistance and inflammation, suggesting that vascular insulin resistance and inflammation are secondary to oxidative stress particularly in the lung.

Table S1: RT-PCR primer.

	**forward primer (5′- -3′):**	**reverse primer (5′- -3′):**
TNF-α	GCATGATCCGCGACGTGGAA	AGATCCATGCCGTTGGCCAG
MIP-1α	ACTGACCTGGAACTGAATGCCTGA	ATGTGGCTACTTGGCAGCAAACAG
MCP-1	ATGCAGGTCCCTGTCATG	GCTTGAGGTGGTTGTGGA
IL-1β	CTCCATGAGCTTTGTACAAGG	TGCTGATGTACCAGTTGGGG
leptin	AAAGAACCTGAGCTGAGGGTGACA	ATGCTAATGTGCCCTGAAATGCGG
adiponectin	AGACCTGGCCACTTTCTCCTCATT	AGAGGAACAGGAGAGCTTGCAACA
PPARγ	ACATAAAGTCCTTCCCGCTGACCA	AAATTCGGATGGCCACCTCTTTGC
SOD1	GATGAAGAGAGGCATGTTGGA	TGTACGGCCAATGATGGAATG
SOD2	GCGGTCGTGTAAACCTCAT	CCAGAGCCTCGTGGTACTTC
SOD3	CTGAGGACTTCCCAGTGAC	GGTGAGGGTGTCAGAGTGT
Catalase	AGCGACCAGATGAAGCAGTG	TCCGCTCTCTGTCAAAGTGTG
HO-1	CACGCATATACCCGCTACCT	CCAGAGTGTTCATTCGAGA
Nrf2	CTCGCTGGAAAAAGAAGTG	CCGTCCAGGAGTTCAGAGG
GSTA^{4}(α)	TGATTGCCGTGGCTCCATTTA	CAACGAGAAAAGCCTCTCCGT
GSTM$^{4.1}$(μ)	AGCTCACGCTATTCGGCTG	GCTCCAAGTATTCCACCTTCAGT
GSTP^{1}(π)	ATGCCACCATACACCATTGTC	GGGAGCTGCCCATACAGAC
GAPDH	AGGTCATCCCAGAGCTGAACG	GGAGTTGCTGTTGAAGTCGCA

Abbr.: IL-1β, interleukin-1β; IL-6, interleukin-6; MCP-1, monocyte chemotactic protein-1; TNF-α, tumor necrosis factor-α; SOD1; soluble superoxide dismutase 1; SOD2, mitochondrial superoxide dismutase 2; SOD3, extracellular superoxide dismutase 3, ecSOD; HO-1, heme oxygenase-1; Nrf2, nuclear factor (erythroid-derived 2)-like 2; GST-α, Glutathione S-transferase alpha; GST-μ, Glutathione S-transferase mu; GST-π, Glutathione S-transferase pi.

Table S2: CAP exposure concentrations, enrichment factors, sizes and elemental compositions of the exposures in Study I-V.

	Study I	Study II	Study III$_{n=2}$	Study IV$_{n=2}$	Study V$_{n=2}$
CAP concentration ($\mu g/m^3$)	87.2	118.1	102.8 (101.5/ 104.1)	44.3 (51.4/37.2)	41.9 (31.9/51.9)
Enrichment factor	8.0	8.0	9.8 (10.9/8.7)	9.0 (9.3/8,9)	6.0 (5.8/6.1)
Mean ± SD (daily median diameter, µm)	0.25±0.01	0.20±0.01	0.23±0.01 (0.22±0.01 /0.26±0.03)	0.24±0.01 (0.23±0.02 /0.25±0.01)	0.22±0.01 (0.27±0.01 /0.20±0.01)
Element composition of CAP, % of total					
S	57.3	53.3	55.5	60.1	59.9
Fe	12.0	8.9	12.1	9.2	8.9
Na	10.8	3.5	4.6	4.5	4.8
Si	4.7	15.7	3.1	6.6	7.6
K	4.5	3.5	3.9	4.2	3.4
Al	0.0	6.0	0.0	2.3	1.5
P	0.0	0.0	0.8	0.0	0.1
Ca	5.9	4.9	10.1	6.9	3.6
Zn	0.7	0.8	0.8	1.0	1.3
Cu	0.6	0.5	0.57	0.9	0.9
others	3.5	2.9	8.6	3.7	7.9

The mean CAP concentration and enrichment factor were determined gravimetrically from the Teflon filters used to collect ambient $PM_{2.5}$ and CAP during the exposure duration of 9 or 30 days. The mean of the daily CAP median diameter during the 9 or 30 day exposures was calculated from the data collected by real-time monitoring using a nephelometer (DataRAM 4). For analysis of the chemical composition, CAP collected on Teflon filter were analyzed by X-ray fluorescence spectrometry (XRF; EX-6600-AF) on representative filters.

Table S3: Effects of CAP exposure for 30 or 9 days on lung inflammation (Study I, III).

**30 days**	**air**	**CAP**	***p***	**air+HFD**	**CAP+HFD**	***p***
IL1β	1.00±0.08	1.01±0.11	*0.961*	1.00±0.04	0.98±0.05	*0.349*
IL6	1.00±0.06	1.03±0.07	*0.773*	1.00±0.04	0.95±0.04	*0.433*
MCP-1	1.00±0.47	1.77±0.88	*0.450*	1.00±0.38	0.66±0.64	*0.458*
TNF-α	1.00±0.10	0.96±0.10	*0.824*	1.00±0.12	0.79±0.14	*0.313*
**9 days**	**air**	**CAP**	***p***	**air+HFD**	**CAP+HFD**	***p***
IL1β	1.00±0.06	1.03±0.03	*0.678*	1.00±0.04	1.09±0.02	*0.108*
IL6	1.00±0.05	0.95±0.05	*0.458*	1.00±0.03	0.98±0.03	*0.536*
MCP-1	1.00±0.20	1.28±0.26	*0.404*	1.00±0.10	0.70±0.09	*0.417*
TNF-α	1.00±0.14	0.99±0.09	*0.939*	1.00±0.12	1.09±0.09	*0.553*

Levels of mRNA were measured in lungs of control (13% kcal fat) or a high-fat diet (HFD, 60% kcal fat) fed mice exposed for 30 or 9 days to air or CAP. Data are mean ± SE normalized to matched air controls; n = 8. Abbr.: HFD, high fat diet; IL-1β, interleukin-1β; IL-6, interleukin-6; MCP-1, monocyte chemotactic protein-1; TNF-α, tumor necrosis factor-α.

Table S4: Mean CAP concentrations (determined by filter weight) of exposures performed from July 2010 to August 2013 (see Fig. 3B).

#	Exposure	CAP
1	May 2013, 9 day	32 $\mu g/m^3$
2	October 2012, 9 day	37 $\mu g/m^3$
3	May 2012, 9 day	51 $\mu g/m^3$
4	August 2013, 9 day	54 $\mu g/m^3$
5	July 2013, 9 day	84 $\mu g/m^3$
6	September 2010, 30 day	87 $\mu g/m^3$
7	August 2010, 9 day	104 $\mu g/m^3$

Table S5: Vascular effects of 9 or 30 days CAP exposure (Study I and III).

**9 days** (n = 4)	**air**	**CAP**	**air+HFD**	**CAP+HFD**
Phenylephrine (PE)				
Tension (mg)	360±44	615±103†	131±26†	150±63*
Active Stress (mN/mm^2)	4.6±0.7	8.5±1.2*	1.8±0.4*	1.8±0.8*
EC_{50} (nM)	62±13	43±20	80±53	109±66
pD_2	7.24±0.09	7.52±0.22	7.44±0.32	7.17±0.23
High Potassium (HI K^+, 100 mM potassium buffer)				
Tension (mg)	758±37	932±72	458±114*	493±86*
Active Stress (mN/mm^2)	9.4±0.7	13.0±0.3*	6.2±1.7†	6.0±1.1*
**30 days** (n = 6-8)	**air**	**CAP**	**air+HFD**	**CAP+HFD**
Phenylephrine (PE)				
Tension (mg)	506±61	242±54*	148±40*	75±27*
Active Stress (mN/mm^2)	6.0±1.1	3.2±0.9*	2.3±0.8*	1.1±0.4*
EC_{50} (nM)	119±20	200±60	619±443	1120±558
pD_2	6.97±0.07	6.80±0.11	6.76±0.24	6.38±0.24
Thromboxane A_2 analog (U46,619, 100 nM)				
Tension (mg)	1220±96	955±87*	597±84*	586±45*
Active Stress (mN/mm^2)	14.3±2.0	11.0±1.6	8.0±1.6*	7.4±1.0*
Acetylcholine (ACh)				
Relaxation (% U46619)	-40±7	-36±6	-44±6	-48±6
EC_{50} (nM)	1750±571	3160±1220	5230±1650	2650±840
pD_2	5.94±0.15	5.73±0.17	5.56±0.22	5.78±0.18

Vascular reactivity was measured in aortas of control (13% kcal fat) or a high-fat diet (HFD, 60% kcal fat) fed mice exposed for 9 or 30 days to air or CAP. The effective concentration producing 50% response (EC_{50}) was assessed by normalizing cumulative concentration responses to 100%, plotting the response vs. the log $[molar]_{agonist}$, and then interpolating the EC_{50}. The pD_2 represents the $-\log(EC_{50})$. Data are mean ± SE; EC_{50}, effective concentration

producing 50% response; pD_2, -log [EC_{50}]; * $p < 0.05$; † $0.10 > p < 0.05$ vs. air-exposed control group (One Way ANOVA, Holm-Sidak post-hoc test).

Table S6: Effects of CAP exposure for 9 days on lung antioxidant defense.

	**air**	**CAP**	***p***
SOD1	1.00±0.25	1.43±0.23	*0.236*
SOD2	1.00±0.10	2.01±0.20	*0.001*
SOD3	1.00±0.18	1.62±0.19	*0.036*
Catalase	1.00±0.22	1.29±0.28	*0.429*
HO-1	1.00±0.25	0.93±0.15	*0.821*
Nrf2	1.00±0.26	0.83±0.08	*0.564*
GST-α	1.00±0.10	1.89±0.29	*0.028*
GST-μ	1.00±0.21	1.62±0.25	*0.089*
GST-π	1.00±0.27	1.20±0.15	*0.532*

Levels of mRNA were measured in lungs isolated from mice exposed for 9 days to air or CAP. Data are mean ± SE normalized to controls; n = 8. Abbr.: SOD1; soluble superoxide dismutase 1; SOD2, mitochondrial superoxide dismutase 2; SOD3, extracellular superoxide dismutase 3, ecSOD; HO-1, heme oxygenase-1; Nrf2, nuclear factor (erythroid-derived 2)-like 2; GST-α, Glutathione S-transferase alpha; GST-μ, Glutathione S-transferase mu; GST-π, Glutathione S-transferase pi.

Table S7: Systemic effects of CAP exposure for 9 days in WT and ecSOD-Tg mice (Study V).

	WT		ecSOD-Tg	
**Parameter, unit, n**	**air**	**CAP**	**air**	**CAP**
body weight, g , 8 -15	29.02±1.26	28.68±0.54	27.60±0.88	29.68±0.68
Glucose, mg/dL, 4-6	233±28	177±8	212±7	201±8
Insulin, ng/mL, 4-8	0.39±0.04	0.33±0.01	0.45±0.02	0.40±0.03
NO_x, μM, 8-12	11.71±5.23	$3.83 \pm 0.67^{+}$	11.59±6.92	9.05±3.11
heart:bw $x10^3$, 8 -15	5.13±0.18	4.59±0.11	4.83±0.18	4.57±0.08
lung:bw $x10^3$, 8 -15	5.54±0.28	5.36±0.11	5.44±0.07	5.25±0.13
spleen:bw $x10^3$, 8 -15	3.45±0.36	2.93±0.09	3.23±0.17	3.10±0.08

Blood and plasma parameter were measured in WT and ecSOD-Tg mice exposed to air or CAP for 9 days. Plasma insulin levels were determined by ELISA (Mouse Insulin ELISA, ALPCO) and plasma NO_x levels were measured by Griess Assay (Griess Reagent System, Promega). Data are mean ± SE; $^{+}$ $p < 0.1$; air vs. CAP. Abbr.: bw, body weight; ecSOD, extracellular superoxide dismutase.

REFERENCES

Brook RD, Rajagopalan S, Pope CA, 3rd, Brook JR, Bhatnagar A, Diez-Roux AV, et al. 2010. Particulate matter air pollution and cardiovascular disease: An update to the scientific statement from the american heart association. Circulation 121:2331-2378.

Conklin DJ, Haberzettl P, Prough RA, Bhatnagar A. 2009. Glutathione-s-transferase p protects against endothelial dysfunction induced by exposure to tobacco smoke. Am J Physiol Heart Circ Physiol 296:H1586-1597.

Folz RJ, Abushamaa AM, Suliman HB. 1999. Extracellular superoxide dismutase in the airways of transgenic mice reduces inflammation and attenuates lung toxicity following hyperoxia. J Clin Invest 103:1055-1066.

Haberzettl P, Vladykovskaya E, Srivastava S, Bhatnagar A. 2009. Role of endoplasmic reticulum stress in acrolein-induced endothelial activation. Toxicol Appl Pharmacol 234:14-24.

Haberzettl P, Lee J, Duggineni D, McCracken J, Bolanowski D, O'Toole TE, et al. 2012. Exposure to ambient air fine particulate matter prevents vegf-induced mobilization of endothelial progenitor cells from the bone marrow. Environ Health Perspect 120:848-856.

Haberzettl P, Bhatnagar A, Conklin DJ. 2014. Particulate matter and oxidative stress–pulmonary and cardiovascular targets and consequences. Systems Biology of Free Radicals and Antioxidants:1557-1586.

Hellmann J, Tang Y, Kosuri M, Bhatnagar A, Spite M. 2011. Resolvin d1 decreases adipose tissue macrophage accumulation and improves insulin sensitivity in obese-diabetic mice. FASEB J 25:2399-2407.

Lund AK, Lucero J, Lucas S, Madden MC, McDonald JD, Seagrave JC, et al. 2009. Vehicular emissions induce vascular mmp-9 expression and activity associated with endothelin-1-mediated pathways. Arterioscler Thromb Vasc Biol 29:511-517.

O'Toole TE, Hellmann J, Wheat L, Haberzettl P, Lee J, Conklin DJ, et al. 2010. Episodic exposure to fine particulate air pollution decreases circulating levels of endothelial progenitor cells. Circ Res 107:200-203.

Sithu SD, Srivastava S, Siddiqui MA, Vladykovskaya E, Riggs DW, Conklin DJ, et al. 2010. Exposure to acrolein by inhalation causes platelet activation. Toxicol Appl Pharmacol 248:100-110.

Sun Q, Yue P, Deiuliis JA, Lumeng CN, Kampfrath T, Mikolaj MB, et al. 2009. Ambient air pollution exaggerates adipose inflammation and insulin resistance in a mouse model of diet-induced obesity. Circulation 119:538-546.

Wetzelberger K, Baba SP, Thirunavukkarasu M, Ho YS, Maulik N, Barski OA, et al. 2010. Postischemic deactivation of cardiac aldose reductase: Role of glutathione s-transferase p and glutaredoxin in regeneration of reduced thiols from sulfenic acids. J Biol Chem 285:26135-26148.

Wheat LA, Haberzettl P, Hellmann J, Baba SP, Bertke M, Lee J, et al. 2011. Acrolein inhalation prevents vascular endothelial growth factor-induced mobilization of flk-1+/sca-1+ cells in mice. Arterioscler Thromb Vasc Biol 31:1598-1606.

Xu X, Liu C, Xu Z, Tzan K, Zhong M, Wang A, et al. 2011. Long-term exposure to ambient fine particulate pollution induces insulin resistance and mitochondrial alteration in adipose tissue. Toxicol Sci 124:88-98.

Yadav AS, Harris NR. 2011. Effect of tempol on diabetes-induced decreases in retinal blood flow in the mouse. Curr Eye Res 36:456-461.
